# Immunotherapeutic approach to reduce senescent cells and alleviate senescence‐associated secretory phenotype in mice

**DOI:** 10.1111/acel.13806

**Published:** 2023-03-26

**Authors:** Niraj Shrestha, Pallavi Chaturvedi, Xiaoyun Zhu, Michael J. Dee, Varghese George, Christopher Janney, Jack O. Egan, Bai Liu, Mark Foster, Lynne Marsala, Pamela Wong, Celia C. Cubitt, Jennifer A. Foltz, Jennifer Tran, Timothy Schappe, Karin Hsiao, Gilles M. Leclerc, Lijing You, Christian Echeverri, Catherine Spanoudis, Ana Carvalho, Leah Kanakaraj, Crystal Gilkes, Nicole Encalada, Lin Kong, Meng Wang, Byron Fang, Zheng Wang, Jin‐an Jiao, Gabriela J. Muniz, Emily K. Jeng, Nicole Valdivieso, Liying Li, Richard Deth, Melissa M. Berrien‐Elliott, Todd A. Fehniger, Peter R. Rhode, Hing C. Wong

**Affiliations:** ^1^ HCW Biologics Inc. Miramar Florida USA; ^2^ Division of Oncology Washington University School of Medicine St. Louis Missouri USA; ^3^ Department of Pharmaceutical Sciences Nova Southeastern University Fort Lauderdale Florida USA

**Keywords:** aging, cellular immunology, circadian genes, immunotherapy, inflammation, physical performance, senescence, senescent cell reduction, senomorphic, type 2 diabetes

## Abstract

Accumulation of senescent cells (SNCs) with a senescence‐associated secretory phenotype (SASP) has been implicated as a major source of chronic sterile inflammation leading to many age‐related pathologies. Herein, we provide evidence that a bifunctional immunotherapeutic, HCW9218, with capabilities of neutralizing TGF‐β and stimulating immune cells, can be safely administered systemically to reduce SNCs and alleviate SASP in mice. In the diabetic *db/db* mouse model, subcutaneous administration of HCW9218 reduced senescent islet β cells and SASP resulting in improved glucose tolerance, insulin resistance, and aging index. In naturally aged mice, subcutaneous administration of HCW9218 durably reduced the level of SNCs and SASP, leading to lower expression of pro‐inflammatory genes in peripheral organs. HCW9218 treatment also reverted the pattern of key regulatory circadian gene expression in aged mice to levels observed in young mice and impacted genes associated with metabolism and fibrosis in the liver. Single‐nucleus RNA Sequencing analysis further revealed that HCW9218 treatment differentially changed the transcriptomic landscape of hepatocyte subtypes involving metabolic, signaling, cell‐cycle, and senescence‐associated pathways in naturally aged mice. Long‐term survival studies also showed that HCW9218 treatment improved physical performance without compromising the health span of naturally aged mice. Thus, HCW9218 represents a novel immunotherapeutic approach and a clinically promising new class of senotherapeutic agents targeting cellular senescence‐associated diseases.

## Graphical abstract

Bifunctional immunotherapeutic HCW9218 functions as a novel SNC‐reducing and senomorphic agent in mice. Subcutaneous administration of HCW9218 activates NK, innate lymphoid group‐1, and CD8^+^ T cells, and neutralizes TGF‐ß to reduce senescent cells (SNC‐reducing) and SASP (senomorphic) leading to lower chronic inflammation and restored tissue homeostasis.

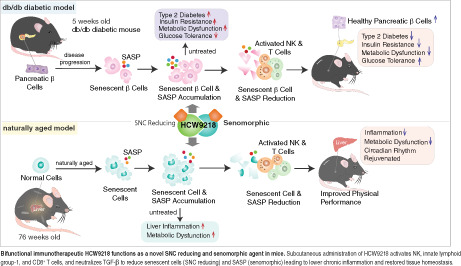

**Abbreviations**
ECAR: extracellular acidification rate
ILCs: innate lymphoid cells
OCR: oxygen consumption rate
SASP: senescence‐associated secretory phenotype
SNCs: senescent cells
TGF‐β: transforming growth factor beta
UMAP: Uniform Manifold Approximation and Projection

## 1 INTRODUCTION

Cellular senescence is a form of irreversible growth arrest accompanied by phenotypic and metabolic changes, resistance to apoptosis, and activation of damage‐sensing signaling pathways. Senescence is considered a stress response that can be induced by a wide range of intrinsic and extrinsic insults, including oxidative and genotoxic stress, DNA damage, telomere attrition, oncogenic activation, mitochondrial dysfunction, or exposure to chemotherapeutic agents (Chandrasekaran et al., 2017; Courtois‐Cox et al., 2008; McHugh & Gil, 2018; Sharpless & Sherr, 2015; Takahashi et al., 2017; Ziegler et al., 2015). This accelerated senescence response, independent of telomere shortening, is known as premature senescence (Serrano et al., 1997). Senescent cells (SNCs) remain metabolically active, and they can influence tissue homeostasis through their senescence associated secretory phenotype (SASP) (He & Sharpless, 2017), which is comprised of a wide range of inflammatory cytokines (IL‐1α, IL‐1β, IL‐6, IL‐8, TNF‐α), growth factors (TGF‐β, PDGF‐AA, insulin‐like growth factor‐binding proteins [IGFBPs]), chemokines (CCL‐2, CCL‐20, CCL‐7, CXCL‐4, CXCL1, and CXCL‐12), and matrix metalloproteinases (MMP‐3, MMP‐9) that operate in a cell‐autonomous manner to reinforce senescence (autocrine effects) and communicate with and modify the microenvironment (paracrine effects) (Milanovic et al., 2018).

Senescence is considered a physiologic process and is important in promoting wound healing (Demaria et al., 2014; Jun & Lau, 2010a), tissue homeostasis (Brighton et al., 2017), regeneration, embryogenesis (Muñoz‐Espín et al., 2013), fibrosis regulation (Jun & Lau, 2010b; von Kobbe, 2018), and tumorigenesis suppression (von Kobbe, 2018). However, accumulation of SNCs also drives aging and age‐related diseases like diabetes, osteoporosis, cardiovascular diseases, dementia, neurodegenerative disorders, renal failure, sarcopenia, and macular degeneration (Bennett & Clarke, 2017; McHugh & Gil, 2018; Triana‐Martinez et al., 2020), while SNC removal improves health span and life span in experimental animal models (Baker et al., 2016; Ogrodnik et al., 2021). Thus, senolytic and senomorphic therapies that clear SNCs and lower SASP with targeted drugs are being vigorously pursued for a healthy longevity (Sedrak et al., 2021; van Deursen, 2019). It has been recently shown that an aged immune system drives senescence and aging of solid organs (Yousefzadeh et al., 2021). Signaling from the SASP transforming growth factor (TGF)‐β is also known to play a major role in cellular senescence and aging‐related pathology (Tominaga & Suzuki, 2019). Therefore, we hypothesized that an immunotherapeutic agent that rejuvenates a dysfunctional immune system and neutralizes TGF‐β would act as an effective SNC reducing and senomorphic drug. Previously, we showed that the bifunctional immunotherapeutic HCW9218, comprising TGF‐β receptor II and IL‐15/IL‐15 receptor α domains, exhibited potent TGF‐β neutralizing and immune cell‐stimulating activities, resulting in enhanced metabolic and cytotoxic activities of immune cells and promoted natural killer (NK)‐cell‐mediated clearance of therapy‐induced senescent (TIS) cells in tumors and normal tissues in vivo (Chaturvedi et al., 2022; Liu et al., 2021). In this study, we employed type‐2 diabetic *db/db* mice, as a model for metabolic‐dysfunction‐induced senescence, and naturally aged C57BL/6J mice for chronological age‐induced cellular senescence, to assess whether HCW9218 can function as an SNC reducing and senomorphic drug under conditions where the stress inducers of cellular senescence are poorly defined or unknown.

## 2 HCW9218 TREATMENT ENHANCES IMMUNE‐MEDIATED BIOLOGICAL ACTIVITIES IN DIABETIC *db/db* MICE

**FIGURE 1:**
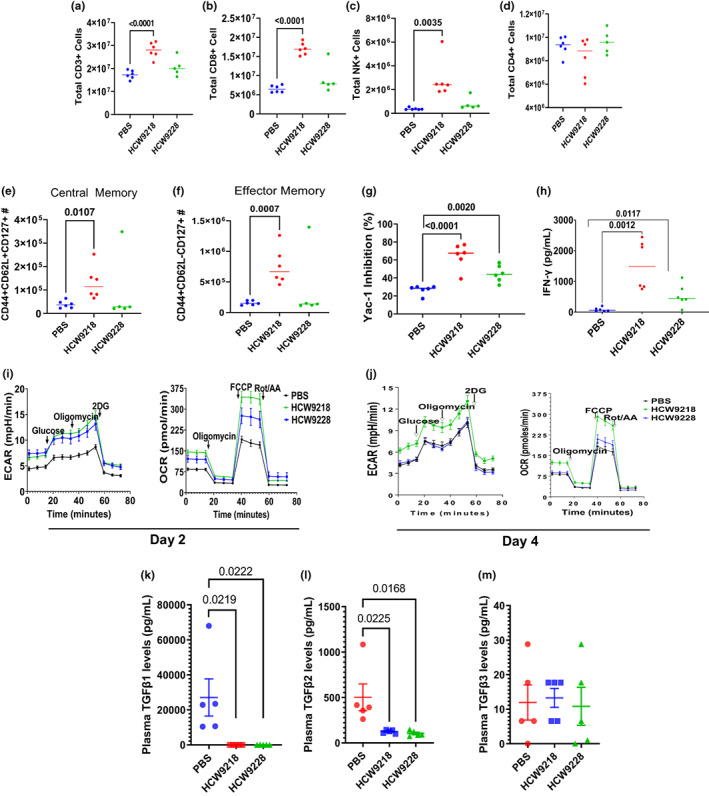
HCW9218 enhances immune‐mediated biological activities in *db/db* micee. (a–d). Representative flow cytometry data showing increase in immune cell surface makers on splenocytes of HCW9218‐treated mice at Day 4 compared to controls. Individual value plot show the mean (*n* = 6/group) from two independent experiments. (e, f) Representative flow cytometry data showing increase in central memory cell and effector memory cell numbers in splenocytes of mice treated with HCW9218 compared to PBS‐treated controls at Day 4. Individual value plot show the mean (*n* = 6/group) from two independent experiments. (g) Killing of Yac‐1 target cells by in vivo HCW9218 treated splenocytes compared to control splenocytes. Individual value plot shows the mean (*n* = 6/group) from two independent experiments. (h) Increase in interferon (IFN)‐γ released by CD3^+^ cells upon antigen‐independent stimulation by in vivo HCW9218 treated and ex vivo α‐CD3/α‐CD28 beads stimulated splenocytes compared to PBS control. Individual value plots show mean (*n* = 6/group) from one experiment. (i, j) Representative data for increase in extracellular acidification rates (ECAR) and oxygen consumption rates (OCR) data from splenocytes of HCW9218, HCW9228 or PBS‐treated mice and analyzed by Seahorse XFe Bioanalyzer (Agilent). (k‐m) ELISA data showing decrease in TGFβ1 and TGFβ2 but not TGFβ3 in plasma after HCW9218 or HCW9228 treatment. Individual value plot show the mean ± SEM (*n* = 5/group) from two independent experiments. *p* values were determined by ordinary one‐way ANOVA with Tukey's multiple comparisons test.

It has been shown that metabolic reprogramming of immune cells in obesity limits antitumor responses (Michelet et al., 2018). Therefore, we first evaluated whether metabolically dysfunctional *db/db* mice retained immune cell stimulatory capability following HCW9218 treatment. Mice were injected subcutaneously with 3 mg/kg of HCW9218 or HCW9228 (a derivative of HCW9218 without IL‐15 activity due to an IL‐15D8N mutation; Liu et al., 2021), or PBS (negative control). Mice treated with HCW9218, but not HCW9228 or PBS, showed higher percentages of CD3^+^ CD8^+^ T cells, and NK cells, but not CD4^+^ T cells, in the spleen (Figure 1a–d). These cells also expressed CD44, CD62L, and CD127 markers of central and effector memory‐cell phenotypes (Figure 1e,f). HCW9218‐treated splenocytes also showed significantly enhanced killing of NK‐sensitive Yac‐1 cells (Figure 1g) and increased interferon (IFN)‐γ released by CD3^+^ cells upon antigen‐independent stimulation (Figure 1h) compared to HCW9228 treatment and PBS control. Since metabolic pathways are linked to immune‐cell fate decision and effector functions (Ganeshan & Chawla, 2014; Jung et al., 2019), we further determined the extracellular acidification rate (ECAR) and oxygen consumption rate (OCR) of splenocytes following HCW9218 treatment in *db/db* mice. Splenocytes from HCW9218‐treated mice on days (D)2 and D4 showed enhanced parameters of glycolysis and mitochondrial respiration (Figure 1i,j, Figure S1A–D). These findings indicate that HCW9218, but not HCW9228 or PBS, was able to stimulate the NK and T cells in an IL‐15 dependent manner in metabolically dysfunctional *db/db* mice. As anticipated, both HCW9218 and HCW9228 significantly reduced plasma levels of TGF‐β1 and TGF‐β2 in treated *db/db* mice (Figure 1k‐m).

## 3 HCW9218 TREATMENT REDUCES SENESCENT PANCREATIC ISLET BETA CELLS AND SASP FACTORS AND IMPROVES TYPE‐2 DIABETES PROFILE OF *db/db* MICE

**FIGURE 2:**
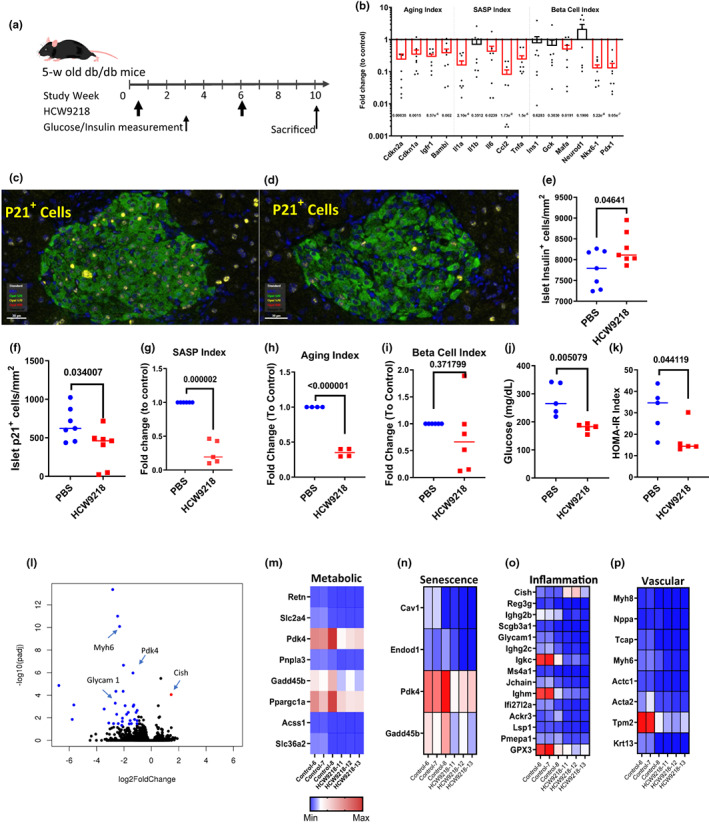
HCW9218 treatment reduces senescent pancreatic islet β cells and SASP factors to improve type‐2 diabetes of *db/db* mice. (a) Schema of HCW9218 treatment in *db/db* mouse model. (b) Expression of Aging, SASP, and β Cell index (related genes) in islet transcript was analyzed by quantitative PCR and normalized to control treatment. Individual value plot shows the mean  (*n* = 7/group) from two independent experiments. (c, d) Immunofluorescent staining of p21^+^ cells (yellow) and insulin^+^ β islet cells (green) in pancreatic tissue sections PBS (c), HCW9218 (d). (e) Number of insulin‐positive islet cells in tissue section. (f) Number of p21^+^ senescent cell in β islet cells in tissue section. (g–i) Relative comparison of Aging, SASP, and Beta Cell index in pancreas from HCW9218‐ and PBS‐treated mice was determined by quantitative PCR. Individual value plot show the mean (*n* = 6/group) from two independent experiments. (j) Fasting blood glucose after HCW9218 treatment. Graph shows mean (*n* = 5/group) from one experiment. (k) HOMA‐IR index after HCW9218 treatment. (l) Volcano plot for RNA‐seq analysis on the livers of *db/db* mice. (m–p) Heat Map for Metabolic, Senescence, Inflammation, and Vascular genes from bulk RNA‐Seq analysis of liver samples. *p* values were determined by Student's *t* tests.

Metabolic dysfunction induces senescence of pancreatic β cells, and removal of these senescent β cells was previously shown to improve glucose metabolism and β cell functions while decreasing expression of markers of aging, senescence, and SASP (Aguayo‐Mazzucato et al., 2019). Thus, reducing the senescent cell burden may potentially alleviate type‐2 diabetes (T2D). To assess whether HCW9218 treatment can remove senescent β cells, 5‐week‐old male *db/db* mice fed with a standard chow diet were subcutaneously administered HCW9218 (3 mg/kg) or PBS (control group), with a second dose 6 weeks later (Figure 2a). qRT‐PCR analysis of the pancreas showed that HCW9218 treatment reduced *Cdkn1a* and *Cdkn2a* expression, which encode cyclin‐dependent kinase (CDK) inhibitors p21 and p16, respectively, as markers and effectors of β‐cell senescence (Figure 2b; Gorgoulis et al., 2019). In addition to *Cdkn1a* and *Cdkn2a*, expression of *Igfr1*, *Bambi*, *Il1a*, *Il6*, *Ccl2*, and *Tnfa* was also lowered in the pancreas of HCW9218‐treated *db/db* mice compared to the PBS control group (Figure 2b). Accumulation of p21^+^ SNCs, which was observed in pancreatic islets of control *db/db* mice, was significantly reduced in HCW9218‐treated mice as evidenced by immunofluorescent staining of pancreatic sections (Figure 2c,d). Insulin‐positive islet β cells were significantly increased (Figure 2e), and p21^+^ cells were significantly reduced in the pancreas (Figure 2f) of the HCW9218 treatment group compared to control mice. Genes associated with the SASP Index and Aging Index were significantly reduced in the pancreas following HCW9218 treatment compared to controls (Figure 2g,h), whereas gene expression of the β cell index was not significantly changed in HCW9218‐ vs. PBS‐treated *db/db* mice (Figure 2i). Collectively, these data suggest that HCW9218 has potent SNC‐ reducing and senomorphic activities to eliminate SNCs and SASP factors in diabetic *db/db* mice. Moreover, we found that fasting glucose levels and the insulin resistance index (HOMA‐IR) were significantly reduced following HCW9218 treatment compared to the PBS group (*p* = 0.0051, Figure 2j and *p* = 0.0441, Figure 2k). These results corroborate previous findings that the reduction of senescent islet β cells improves the healthy life span of T2D mice (Aguayo‐Mazzucato et al., 2019).

Since T2D is a metabolic disease and the liver is a key metabolic organ governing body energy metabolism, we also performed RNA‐Seq analysis on the livers of *db/db* mice following HCW9218 treatment. Of the differentially expressed liver genes, one was upregulated and 32 were downregulated which together could be grouped (by STRING) into four clusters based on function (Figure 2l; Table S1). The expression of 8 genes related to glucose, lipid, or amino acid metabolism was significantly reduced in the liver following HCW9218 treatment (Figure 2m). Among this group, HCW9218‐mediated downregulation of resistin (*Retn*), previously found to be mainly synthesized in adipocytes, was particularly interesting. Resistin has been shown to induce insulin resistance in mice partially through the toll‐like receptor 4 signaling pathway (Benomar & Taouis, 2019; Shi, Fan, et al., 2019; Shi, Hua, et al., 2019), and its downregulation by HCW9218 treatment could contribute to the reduction of insulin resistance (Figure 2m). The expression of cellular senescence related genes, *Cav1*, *Endod1*, *Pdk4*, and *Gadd45b*, was also downregulated (Figure 2n) suggesting that HCW9218 treatment reduces senescent cell levels in the liver. Fourteen proinflammatory genes were downregulated and one gene was upregulated (*Cish*) (Figure 2o), suggesting that HCW9218 treatment reduced liver inflammation. As a negative regulator of IL‐15 (Delconte et al., 2016), *Cish* upregulation by HCW9218 treatment is expected and indicates that HCW9218 activated the liver immune cells in *db/db* mice. The expression of eight genes related to vascular regulation was also reduced (Figure 2p). Since diabetes is known to induce vascular dysfunction and cellular senescence is implicated in this pathological process (Paneni et al., 2013; Yin & Pickering, 2016), this result further suggests that the reduction of SNCs and SASP in *db/db* mice could favorably impact vascular health in diabetes. Collectively, the results of this RNA‐Seq analysis support the hypothesis that HCW9218 treatment reduces the cellular senescence, SASP, and gluconeogenesis induced by metabolic dysfunction to improve glucose metabolism, metabolic homeostasis, and lower sterile inflammation in the livers of T2D *db/db* mice.

## 4 HCW9218 STIMULATES IMMUNE CELL ACTIVITIES AND METABOLIC FUNCTIONS WHILE REDUCING SASP AND CELLULAR SENESCENCE MARKERS IN NATURALLY AGED MICE

Although HCW9218 treatment has been shown to effectively reduce therapy‐induced and metabolic dysfunction‐induced SNCs and SASP in vivo (Chaturvedi et al., 2022), it is unknown whether HCW9218 could also eliminate the heterogeneous population of SNCs generated and accumulated during natural aging. The heterogeneity of accumulated SNCs in the aging process is the result of poorly defined cell and tissue context‐dependence inducers over time (Calcinotto et al., 2019). Thus, we evaluated both SNC‐ reducing and senomorphic activities of HCW9218 in naturally aged mice.

**FIGURE 3:**
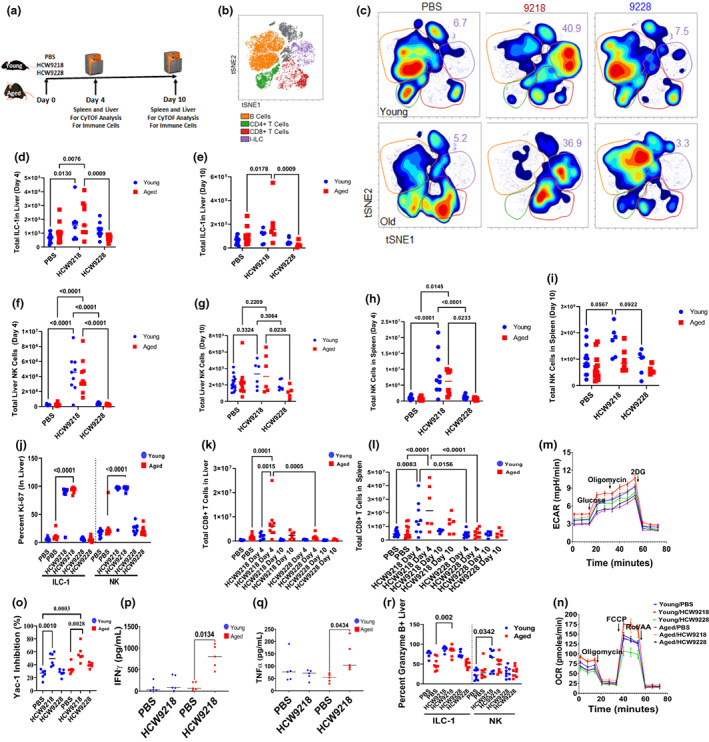
HCW9218 stimulates immune cell activity and metabolic functions in liver of naturally aged mice. (a) Schema of HCW9218 and HCW9228 treatment in young and aged mice model for Day 4 and Day 10. (b) Composite unbiased t‐SNE identifying B cells, T cells, and group 1 ILCs. (c) Representative viSNE plots colored by density from livers harvested at Day 4. Gates are colored by population and inset numbers represent frequency of I‐ILC (ILC1 cells). (d, e) Summary data from three independent experiments, (*n* = 6/group) demonstrating the increased total ILC‐1 cell frequency in the liver at Day 4 (d) and Day 10 (e). (f–i) Total NK cell frequency in the liver at Day 4 (f) and Day 10 (g) and in the spleen at Day 4 (h) and Day 10 (i) relative to control. (j) Percentage positive of Ki‐67 proliferation markers in liver. (k, l) Total CD8^+^ T‐cell frequency in the liver (k) and spleen (l) at Day 4 and Day 10. (m, n) Representative data for increase in extracellular acidification rates (ECAR) (m) and oxygen consumption rates (OCR) (n) data from splenocytes stimulated in vivo with HCW9218 from young and aged mice by Seahorse XFe bioanalyzer compared to control. (o) Measuring the ex vivo cytotoxic activity on Yac‐1 target cells by in vivo HCW9218‐stimulated splenocytes from young and aged mice compared to controls (PBS or HCW9228) by flow cytometry. Individual value plot shows the mean (*n* = 6/group) from two independent experiments. (p, q) Increase in IFN‐γ and TNF‐α released by CD3^+^ cells upon antigen‐independent stimulation by in vivo HCW9218 treated and ex vivo α‐CD3/α‐CD28 beads stimulated splenocytes compared to control aged and young mice measured by MAGPIX multiplexing system. Individual value plot show the mean (*n* = 5/group) from two independent experiments. (r) Representative flow cytometry data showing increase in percentage of intracellular Granzyme B markers in liver ILC‐1 and NK cells in young and aged mice treated with HCW9218 or controls. Individual value plot shows the mean (*n* = 6/group) from two independent experiments. *p* values were determined by ordinary one‐way ANOVA with Tukey's multiple comparisons test.

First, we evaluated whether HCW9218 exhibited immune cell stimulatory activity in aged mice which are reported to exhibit immunosenescence (Budamagunta et al., 2021). To determine the impact of HCW9218 on the immune cells of aged and young mice, we performed mass cytometry utilizing an antibody panel (Table S3) that focuses on lymphocytes (B cells, CD4^+^, CD8^+^ T cells) and group 1 innate lymphoid cells (ILC‐1s) (NK cells and ILC1s). After a single injection of PBS (vehicle control), HCW9218, or HCW9228 (IL‐15 negative control), mononuclear cells from the liver and spleen were evaluated on either D4 or D10 after treatment (Figure 3a). Unbiased t‐SNE‐based clustering identified lymphocyte annotated subsets (Figure 3b), and density maps demonstrated a marked alteration in immune cell composition on D4 (Figure 3c). In the liver, HCW9218 treatment significantly increased group 1 innate lymphoid cells (Figure 3d,e) and NK cells (Figure 3f,g) on D4 and D10 in both aged and young mice. In the spleen, however, total NK cells significantly increased in both aged and young mice on D4 but not on D10 (Figure 3h,i). However, HCW9228 did not induce any activation in NK or ILC‐1 cells. Both liver NK cells and ILC‐1s showed evidence of HCW9218‐mediated activation with increased expression of Ki67 in the aged mice (Figure 3j). In addition, total CD8^+^ T cells in the liver and spleen of aged mice significantly increased on D4 following HCW9218 treatment compared to controls (Figure 3k,l). Liver CD4^+^ T cells in aged and young mice also increased on D4 following HCW9218 treatment (Figure S2J). There was no effect of HCW9218 on Treg frequency (Figure S2K). These findings were further confirmed by flow cytometry analysis on immune cells from the spleen and peripheral blood of HCW9218‐treated young and aged mice (Figure S2A–G).

Splenocytes from both young and aged mice treated with HCW9218 showed elevated glycolysis (Figure 3m and Figure S3A), mitochondrial respiration rates (Figure 3n and Figure S3B), and enhanced killing of NK‐sensitive Yac1 cells (Figure 3o). Splenocytes from aged mice showed increased IFNγ (Figure 3p) and TNFα (Figure 3q) released by CD3^+^ cells upon anti‐CD3/anti‐CD28 stimulation. The liver ILC‐1 (Aged mice) and NK (Young mice) granzyme B levels (Figure 3r) were also upregulated by HCW9218 treatment.

Collectively, these findings provide evidence that HCW9218 treatment effectively stimulates and promotes the proliferation of NK cells and CD8^+^ T cells and improves the fitness (i.e., metabolic functions) of these immune cells in lymphoid tissue and liver of naturally aged mice.

## 5 HCW9218 REDUCES SNCs AND SASP IN PERIPHERAL ORGANS IN NATURALLY AGED MICE

**FIGURE 4:**
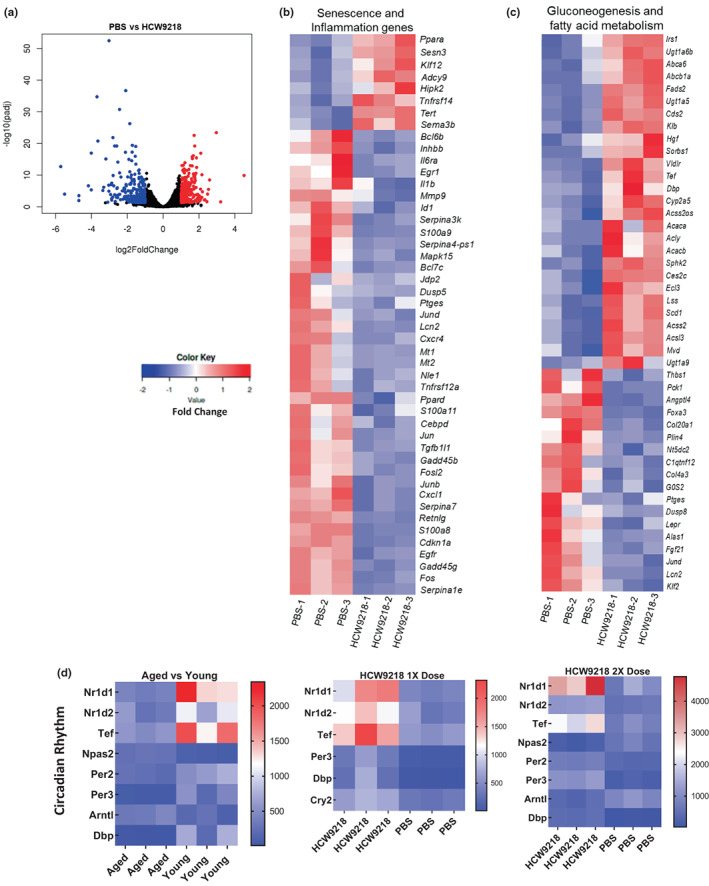
HCW9218 reduces inflammation (SASP) and cellular senescence markers of naturally aged mice in liver after one or two subcutaneous doses of HCW9218 or PBS. (a) HCW9218 treated aged mice liver cells bulk RNA‐Seq difference compared to control treatment in volcano plots after 60 days. (b, c) Heat maps of the differentially expressed senescence and inflammation, glucogenesis and fatty acid metabolism in liver after treatment with HCW9218 compared to control treatment by bulk RNA‐Seq plotted in fold change values (adjusted *p* value <0.05). (d) Heat maps of the differentially expressed circadian rhythm associated genes in liver after treatment with HCW9218 compared to control treatment by bulk RNA‐Seq. Scale shows the minimum and maximum normalized genes hit counts (adjusted *p* value <0.05).

We next examined long‐term changes in the expression of inflammation and senescence‐associated genes in aged mice (76 weeks) receiving either one or two subcutaneous doses of HCW9218 (3 mg/kg) or PBS (control). RNA‐Seq analysis was performed on the liver isolated at 60 or 90 days after HCW9218 treatment to determine global changes in transcription. Significant differentially expressed genes were clustered by gene ontology, and enrichment of gene ontology terms was tested using the Fisher exact test (GeneSCF v1.1‐p2). The liver of HCW9218‐treated aged mice showed dramatic changes in gene expression with 539 differentially expressed mRNAs compared to PBS‐treated mice (Figure 4a). Significant downregulated (e.g., *Cdkn1a*, *Nle1*, *Jund*, *Bcl6*, *Bcl7c*, *and Gadd45β*) or upregulated (e.g., *Tert and Sema3b*) expression of senescence and inflammation associated (SASP) genes (e.g., cytokines: *Il6rα*, *Il1α*, *Il6*, *Tnfα*, *S100a8*, *S100a9*, *S100a11*, *Lcn2*, *Retnlg*, *Inhbb*; chemokines: *Cxcl1*, *Cxcr4*, *Mt1*, and *Mt2*; metalloproteinases: *Mmp9*; gene expression and signaling pathways: e.g., *Cebpd*, *Klf12*, *Egr1*, *Egfr*, *Gadd45β*, *Gadd45g*, *Pparα*, *Pparδ*, *Fos*, *Fosl2*, *Jun*, *Junb*, *Mapk15*, *Adcy9*) was observed following HCW9218 treatment (Figure 4b). HCW9218 treatment also upregulated important gene families associated with immune function (e.g., *Sucnr1*, *Prom1*, *Ascc3*, *Lyst*, *and Sesn*) and reduced transcripts involved with immune suppression (*S100a8*, *S100a9*, *Slfn4*, *Tcim*, *Lcn2*) (Figure S4). Expression of gene sets associated with glucose and fatty acid metabolism (e.g., *Angptl4*, *Gos2*, *C1qtnf12*, *Fads2*, *Sorbs1*, *Zbtb16*, *Mvd*, *Scd1*, *Acaca*, *Acacb*, *Abcb1c*, *Abca6*, *Acly*, *Eci3*, *Ugt1a5*, *Ugta6*, *Ugta9*, *Acsl3*, *Lss*, *Acss2os*, *and Plin4*), fibrosis (e.g., *Col4a3*, *Col20a1*, *Jund*, *and Thbs1*), and other liver functions (e.g., *Dbp*, *Tef*, *and Acss2*) was also altered with HCW9218 treatment (Figure 4c). Unexpectedly, the RNA‐seq analysis also found that transcripts of circadian molecular clock repressor genes (orchestrating circadian rhythms) were altered 60 days after HCW9218 treatment (Figure 4d). Expression of circadian repressor genes *Per*, *Cry*, *Nr1d1*, *Nr1d2*, and *Dbp* was upregulated by the HCW9218 treatment (Figure 4d). 60 days after two‐dose HCW9218 treatment, the repressor genes were found to be upregulated similarly to a single‐dose treatment, but expression of the activator genes *Arntl* and *Npas2* was downregulated (Figure 4d). Remarkably, HCW9218 treatment as either a single‐ or double‐ dose regimen, appeared to reverse the expression pattern of these key circadian‐rhythm genes in aged mice to that of young mice, measured 60 days after treatment (Figure 4d).

**FIGURE 5:**
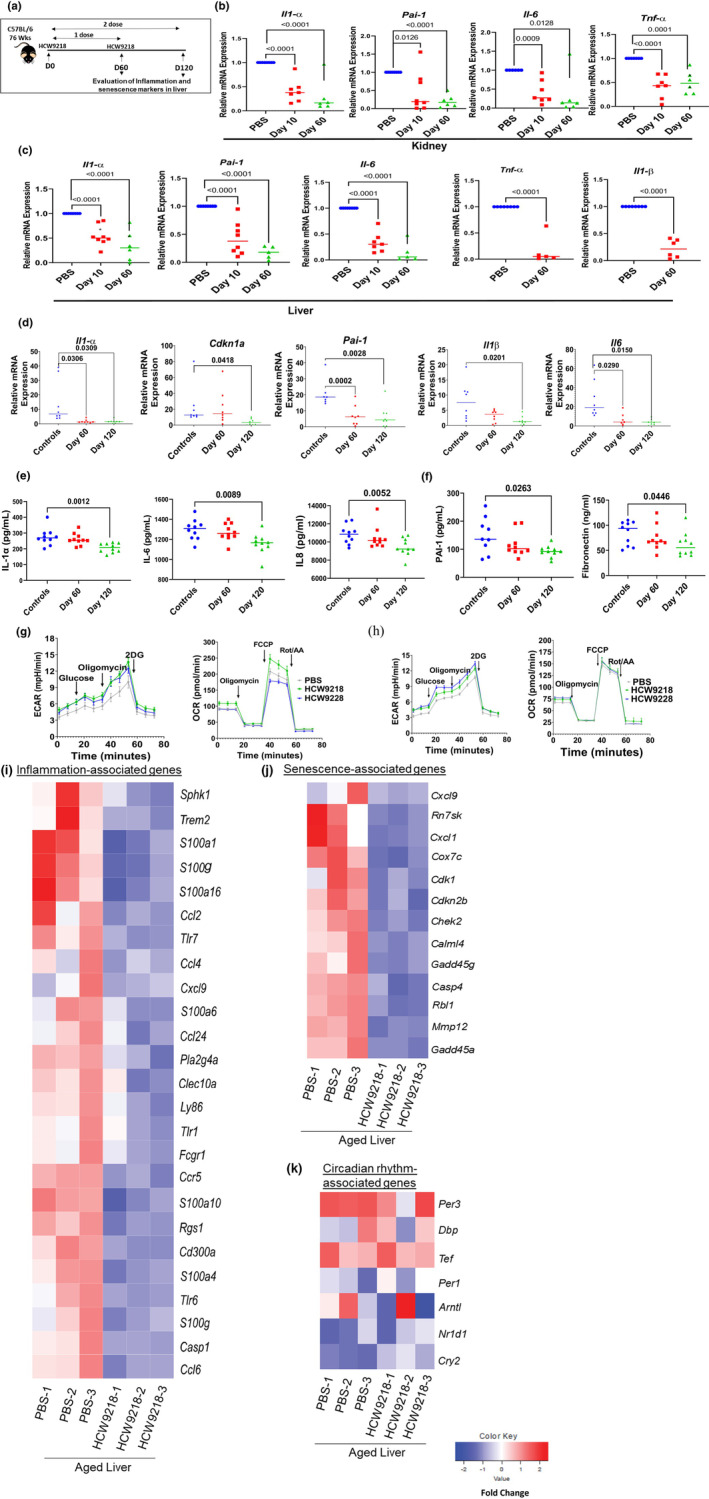
Two‐dose HCW9218 stimulates metabolic functions and reduces inflammation (SASP) and cellular senescence markers in naturally aged mice liver for an extended time. (a) Schema of two‐dose HCW9218 treatment in naturally aged (76 weeks) female C57BL/6 mice that were subcutaneously injected with 3 mg/kg of HCW9218 (*n* = 5–8) or saline. Mice received second dose of HCW9218 at Day 60 and were euthanized at Day 120. (b, c) Relative mRNA expression of *Il1*α, *Pai‐1*, *Il6*, *and Tnf*α in kidney and *Il1β, Il1α, PAI‐1*, *Il6*, *and Tnfα* in liver was analyzed by quantitative PCR after treatment with HCW9218 compared to control at Day 10 and/or Day 60. Individual value plot shows the mean from two independent experiments. (d) Relative mRNA expression *Il1α Cdkn1a*, *Pai‐1*, *Il1b*, and Il6 in liver after treatment with HCW9218 one or two doses compared to control at Day 120 determined by quantitative PCR. Individual value plot shows the means  (*n* = 8/group). (e, f) ELISA data showing protein levels of IL‐1α, IL‐6, IL‐8, PAI‐1, and fibronectin in liver tissue by ELISA liver after treatment with HCW9218 one or two doses compared to control at Day 120. Individual value plot show the mean of 10 mice per group in an experiment. (g, h) Representative data for the increase in extracellular acidification rates (ECAR) and oxygen consumption rates (OCR) data from splenocytes stimulated in vivo with day 60 (g) and day 120 (h) measured by Seahorse XFe bioanalyzer compared to control. *p* values were determined by Student's *t* tests for two group comparison and *p* values were determined by ordinary one‐way ANOVA with Tukey's multiple comparisons test where there are more than two groups. (i‐k) Two‐dose HCW9218 stimulates metabolic functions and reduces inflammation (SASP) and cellular senescence markers in naturally aged mice liver for extended time by bulk RNA‐Seq analysis. Heat maps of the differentially expressed inflammation (i), senescence (j), and circadian rhythm (k) associated genes in liver after treatment with HCW9218 compared to control treatment plotted in fold changes (adjusted *p* value <0.05).

We further analyzed the impacts of HCW9218 treatment on cellular senescence and SASP in peripheral organs of aged mice using qRT‐PCR and ELISA studies (Figure 5a). qRT‐PCR analysis of kidney (Figure 5b) and liver (Figure 5c) of aged mice either 10 or 60 days after a single‐dose HCW9218 treatment showed a significant reduction in gene expression for the signature cellular senescence and SASP genes, *Pai1*, *Il1a*, *Il6*, *Il1b*, and *Tnfa* compared to PBS‐treated control mice. Two‐dose HCW9218 treatment (Figure 5d) also significantly reduced *Il1a*, *Cdkn1a*, *Pai1*, *Il1b*, and *Il6* transcripts in the liver at 120 days post‐treatment initiation compared to the control group. Reduction of IL‐1α, IL‐6, and IL‐8 in the liver was also confirmed at protein levels by ELISA (Figure 5). In a two‐dose treatment regimen, we also found that HCW9218 lowered biomarkers PAI‐1 and fibronectin (Figure 5f), suggesting that HCW9218 could reduce liver fibrosis in aged mice, consistent with significant downregulation of *Col4a3* and *Col20a1* expression observed in the above RNA‐seq study. In addition to gene expression signatures in solid peripheral organs, we also found a long‐lasting impact of HCW9218 on enhancing splenocytes' glycolysis and mitochondrial respiration, which continued 45 days after treatment with the second dose (Figure 5g,h, Figure S5A,B).

The durability of the SNC‐reducing and senomorphic activities of HCW9218 treatment on gene expression was further evaluated using bulk RNA‐seq analysis of liver from aged mice isolated at 120 days. Remarkably, we continued to observe significant downregulation (e.g., *Cdkn1a*) or upregulation (e.g., *Tert*) of senescence and inflammation associated (Figure 5i,j and Table S2) genes (e.g., cytokine: *Il7*, *Il15*, *Il18*, *S100a1*, *S100a4*, *S100a6*, *S100a10*, *S100a16*, *S100g*; chemokines: *Ccl2*, *Ccl4*, *Ccl6*, *Ccl7*, *Ccl8*, *Ccl9*, *Ccl24*, *Ccl25*, *Ccl27*, *Cxcl1*, *Cxcl10*, *Cxcl11*; metalloproteins: *Mmp12*, *Mmp13*, *Mmp27*; gene expression and signaling pathways: *Klf1*, *Klf3*, *Klf7*, *Klf9*, *Klf13*, *Egr1*, *Pparα*, *Jun*, *Fosl2*; *Mapk3*, *Mapk6*, *Mapk7*, *Mapk9*, *Mapk12*, *Mapk15*, *Adcy1*, *Adcy3*, *Adcy5*, *Adcy6*, *Adcy9*, *Adcy10*), and genes associated with metabolic functions (e.g., *Dbp and Tef*) and immune stimulation (e.g., *Lyst*, *Sesn2*, *and Sesn3*) following HCW9218 treatment (Table S2). RNA‐seq analysis also revealed that transcripts of circadian molecular clock repressor genes, *Per1*, *Per3*, *Cry2*, *Nr1d1*, and *Dbp*, were still upregulated 120 days after HCW9218 treatment (Figure 5k). However, following 120 days after two‐dose HCW9218 treatment, the expression of the activator gene *Arntl* was upregulated and the effects on *Npas2* expression became insignificant compared with the HCW9228 treatment (Figure S6B).

To further evaluate whether the TGF‐βRII component of HCW9218 exhibited SNC‐reducing and senomorphic functions, we treated young mice with a single‐dose of HCW9228 and conducted the RNA‐Seq analysis on the livers 10 days after treatment. As shown (Figure S6A), HCW9228 treatment significantly lowered expression of *Cdkn1a* and many circadian clock genes in the liver.

A comparison of the impacts of HCW9218 and HCW9228 120 days after treatment was also performed (Figure S6B). RNA‐seq analysis of liver from treated mice showed that HCW9218, but not HCW9228, maintained the downregulation of *Cdkn1a* expression (Figure S6B), while both treatments continued to upregulate *Tert* gene expression compared with PBS treatment. Interestingly, HCW9228 treatment significantly increased circadian molecular clock activator genes *Arntl* and *Npas2* compared to HCW9218‐treated or the control group (Figure S6B). Since HCW9228 did not activate or promote the proliferation of immune cells (Figure S2A,B), this suggests that direct neutralization of TGF‐β by the TGFβRII component of HCW9218 may contribute to SNC‐ reducing and senomorphic activities of HCW9218 and that the IL‐15 component of HCW9218 provides long‐lasting SNC‐reducing activity.

Taken together, our data indicate that HCW9218 treatment durably reduces genes associated with SNCs and SASP and enhances immune‐cell activities in naturally aged mice. It also suggests that HCW9218 treatment improves metabolic function, fibrosis, and circadian rhythms in liver cells of naturally aged mice.

**FIGURE 6:**
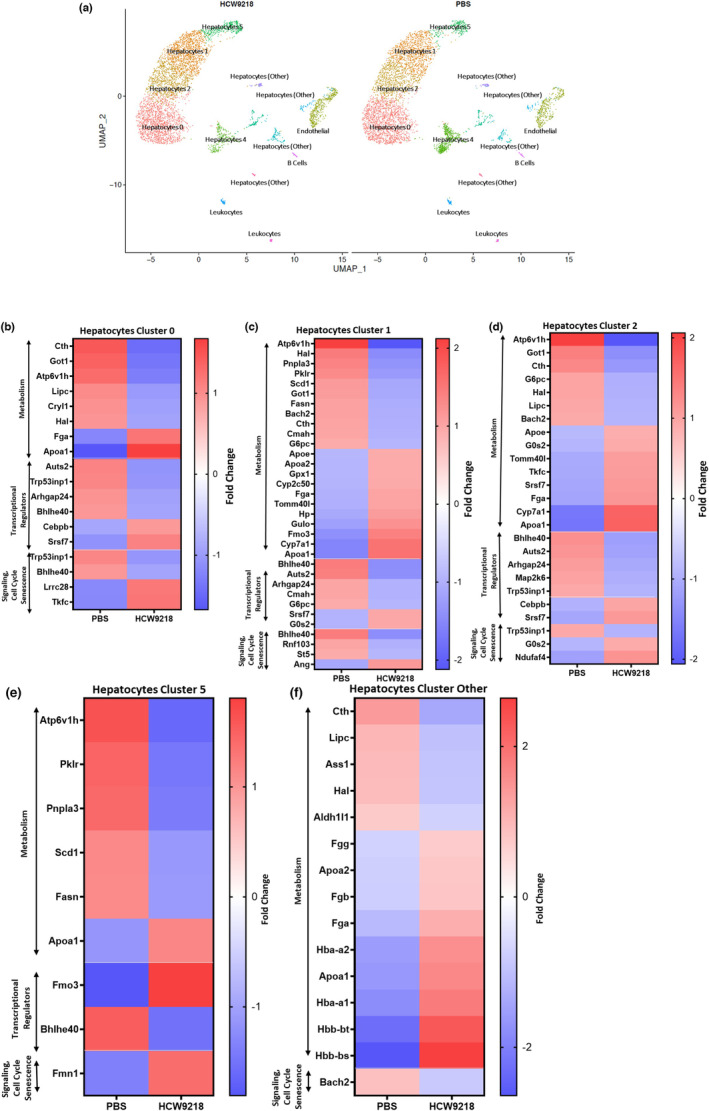
Uniform Manifold Approximation and Projection (UMAP) of single‐cell RNA‐Sequencing data with Seurat analysis. (a) Cells are clustered in 2 dimension using the UMAP dimensionality reduction technique and annotated by different cell types of liver cells after two‐dose treatment of either PBS or HCW9218. (b‐f) Heat maps of the differentially expressed metabolism associated, transcriptional regulators and signaling, cell cycle and senescence associated genes in different clusters of hepatocyte in liver after treatment with HCW9218 compared to control treatment plotted in fold changes (adjusted *p* value <0.05) Uniform Manifold Approximation and Projection (UMAP) of single‐cell RNA sequencing data with Seurat.

To further examine the long‐term impacts of HCW9218 treatments on hepatocyte from naturally aged mice, liver tissues obtained from mice collected 60 days after the second treatment were subjected to single‐cell RNA sequencing (scRNA‐Seq). Computational Seurat analysis of scRNA‐Seq data identified 13 different clusters associated with either PBS or HCW9218 treatment. Uniform Manifold Approximation and Projection (UMAP) analysis detected five major hepatocyte clusters (labeled 0, 1, 2, 4, 5) and four minor clusters (including endothelial, B cells, leukocytes, and other hepatocyte; Figure 6a–c, and Figure S7A–D). For hepatocyte cluster 0, HCW9218 treatment significantly changed the expression of genes involved in metabolism (*Apo1a* and *Tkfc* were upregulated, and *Atp6v1h*, *Cth*, *Got1*, *and Lipc* were downregulated; Figure 6b), transcriptional regulators (*Srsf7* and *Cebpb* were upregulated; and *Auts2*, *Bhlhe40* and *Trp53inp1* were downregulated; Figure 6b), signaling (*Apola* and *Lrrc28* were upregulated), cell cycle and senescence‐associated genes (*Trp53inp1* was downregulated; Figure 6b). The downregulation of *Trp53inp1* and *Bhlhe40* by HCW9218 treatment is particularly interesting because of their role in maintaining p53 stability and the fate of inflammatory and anti‐inflammatory switching (Shahbazi et al., 2013; Yu et al., 2018), respectively, which could result in lowering the expression of *Cdkn1a* (cellular senescence) and SASP in this hepatocyte subset. For hepatocyte cluster 1, HCW9218 treatment not only significantly changed the expression of genes identified in cluster 0, but also detected other genes associated with metabolism (*Fmo3*, *Hp*, *Gpx1*, *Tomm40l*, *Apoa2*, and *Apoe* were upregulated; *Pnpla3*, *Pklr*, *Scd1*, *Bach2*, *Cmah*, and *Fasn* were downregulated; Figure 6c), and signaling (*Ang* was upregulated; *Bach2* and *St5* were downregulated; Figure 6c). In addition, HCW9218 treatments significantly impacted the expression of genes associated with the pathways of cellular senescence such as *NFκB* (*Fmo3*) and *Akt–mTOR* (*Ang*, *Scd‐1*), the stress‐induced cellular senescence (Lin et al., 2021; Manevski et al., 2020) and the anti‐oxidative stress response (*Gpx1*, *Ang*; see hepatocyte cluster 1, Figure 6c). Hepatocyte cluster 2 identified genes associated with the metabolic pathway were *Atp6v1h*, *Cth*, *G6pc*, *Got1*, *Hal*, *Bach2*, and *Lipc* genes which were downregulated by HCW9218 whereas the cholesterol metabolism genes *Apoa1*, *Apoe*, *Cyp7a1*, and *Tomm40l* were upregulated by HCW9218. In addition, the PAR signaling pathway genes *Apoa1* and *Cyp7a1* were found upregulated by HCW9218. Similarly, *Srsf7*, *G0s2*, *Tkfc*, and *Cebpb* involved in transcriptional regulation were upregulated, whereas the *Auts2*, *Bhlhe40*, *Map2k6*, *Arhgap24*, and *Trp53inp1* genes were downregulated by HCW9218 (Figure 6d). Genes grouped in the cluster 2 also overlapped with genes identified the hepatocyte cluster 0 and cluster 1 (Figure 6b,c). There was no significant difference in expression between genes detected in the hepatocytes cluster 4 for HCW9218 and PBS control (Figure 6a). However, in hepatocyte cluster 5 (Figure 6e), the metabolism associated gene *Apoa1* and the pathways of cellular senescence related gene *Fmo3* were upregulated by HCW9218, while genes associated with liver metabolism (*Atp6v1h*, *Fasn*, *Pnpla3*, *Pklr*, and *Scd1*) were downregulated by HCW9218 treatment. In other hepatocyte clusters, the transcriptional repressor gene *Bhlhe40* was also downregulated in hepatocyte cluster 5 (Figure 6e). Further analysis of the remaining small population of hepatocyte clusters as a group identified that the genes associated with metabolic pathways (*Cth*, *Ass1*, *Aldh1l1*, *Hal*, *Bach2*, and *Lipc*) and signaling (*Bach2*) were downregulated whereas the cholesterol metabolism associated genes *Apoa1* and *Apoa2* were upregulated by HCW9218 as compared to PBS treatment (Figure 6f). Expression of genes detected in the endothelial cell cluster (Figure S8A–C) and the leukocyte cluster (data not shown) was not significantly changed by HCW9218 after 60 days of treatment.

Thus, this single‐nucleus RNA‐Seq analysis revealed that HCW9218 treatments impacted the long‐term transcriptomic landscape of hepatocyte subtypes in extensively modulating the transcriptional regulators, metabolism, signaling, cell‐cycle, and senescence pathways in the naturally aged mice. The scRNA‐Seq data are consistent with the RNA‐Seq and qRT‐ PCR data analysis described above. These results indicate that HCW9218 acts as a potent immunotherapeutic senomorphic and SNC‐reducing agent for reducing SASP and clearing senescent cells, respectively.

## 6 HCW9218 AND HCW9228 SUPPORT THE MAINTENANCE OF PHYSICAL PERFORMANCE OF NATURALLY AGED MICE

**FIGURE 7:**
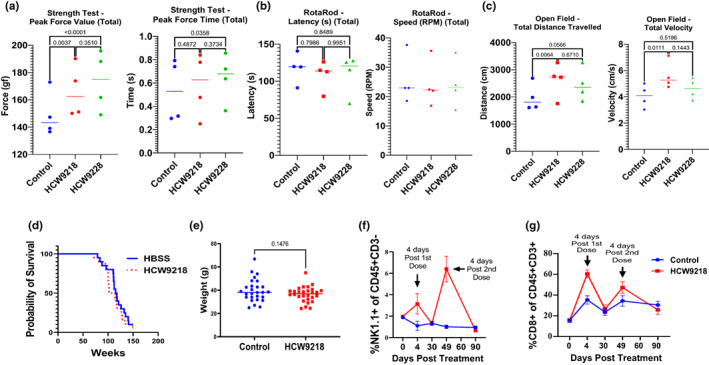
HCW9218 treatment provides significant maintenance of physical performance and was well tolerated by aged mice. (a) Grip strength test performed in aged mice treated with HCW9218 and HCW9228 compared to controls. Data show maintenance of grip strength (peak force) over time using Tukey's multiple comparisons test. (b) Rotarod performance in mice treated with saline, HCW9218 and HCW9228. (c) Open field test in the same mice mentioned above to measure total distance travelled (*n* = 5 for Control, HCW9218, and HCW9228; *p* values were determined by two‐way ANOVA with Tukey's multiple comparisons post hoc). (d) Probability of survival‐monitored for survival and analyzed using the log‐rank test. Summary data are from two experiments with *n* = 10 mice per group represented as mean. (e) Weight was measured after 5 months. (f, g) Representative flow cytometry data showing the percentage of NK cells and CD8^+^ T cells in blood of aged mice after HCW9218 or PBS treatment.

Studies have shown that the elimination of accumulated senescent cells can improve the cognitive functions and alleviate sarcopenia of aged mice (Kirkland & Tchkonia, 2017). Thus, we used a battery of behavioral tests, consisting of grip strength, rotarod, and open field tests, to evaluate whether HCW9218 and HCW9228 treatment could support the maintenance of physical performance (i.e., strength, coordination, and ambulation) of naturally aged mice. Aged C57BL/6 mice were either treated with HCW9218, HCW9228, or PBS, and tests were conducted at four time points: 30 days post‐dose 1 (Trial 1), 3–6 days post‐dose 2 (Trial 2), 30 days post‐dose 2 (Trial 3), and 60 days post‐dose 2 (Trial 4). While results showed only a minimal acute effect of HCW9218 and HCW9228 on performance (Figure S9A–C), significant improvements in the maintenance of performance were observed across time for two of the three tests. Grip strength (Figure 7a) indicated a significant treatment effect with both HCW9218 (*p* = 0.0037) and HCW9228 (*p* < 0.0001) treatment maintaining grip strength (peak force) over time, as compared to PBS treatment. There was also a significant improvement in the amount of time that mice treated with HCW9228 maintained the peak grip level versus PBS controls (*p* = 0.0358). The rotarod test, which focused on motor learning and coordination (Figure 7b), showed no significant differences in the ability to remain on the rotating rod (*F*(2, 48) = 02391, *p* = 0.7882) or the maximum rotation speed attained (*F*(2, 48) = 0.08336, *p* = 0.2394) among the treatment groups. Although these results indicated no change in coordination or motor learning capability, all groups improved in performance over time, indicating motor learning capability was similar across groups. In the open field test (Figure 7c), which allowed the mice to freely explore their environment while measuring ambulation, HCW9218 treatment was associated with a significantly improved distance traveled (*p* = 0.0064), while HCW9228 treatment yielded a nearly significant result (*p* = 0.0566) compared to PBS treatment. Likewise, there was a significant effect of HCW9218 treatment, but not HCW9228 treatment, on speed (total velocity; *p* = 0.0111). Our results collectively suggested that both HCW9218 and HCW9228 treatments provided better overall support for neuromuscular and motor performance than PBS over a long period of time in naturally aged mice.

## 7 LONG‐TERM SAFETY AND TOLERABILITY OF HCW9218 IN MICE

Considering the diverse physiological roles of SNCs in tissue homeostasis, the potential adverse effects of their removal must be considered. Thus, we conducted short‐term and long‐term toxicity studies of HCW9218 treatment in mice. Similar to results described previously (Liu et al., 2021), subcutaneous administration of HCW9218 at 5–100 mg/kg in two doses at a 14‐day interval was well tolerated in a GLP toxicity study in C57BL/6 mice with no observed mortality and no test article related changes in clinical signs or clinical pathology.

The activity and tolerability of HCW9218 were then assessed in naturally aged C57BL/6 mice. Mice (76‐week‐old) treated subcutaneously with a single dose of 3 mg/kg HCW9218 (*n* = 20) or PBS (control; *n* = 20) were observed weekly for changes in overall survival and body weight (Figure 7d,e) with no differences. In subsequent studies, 90‐week‐old mice were treated with two subcutaneous 3 mg/kg doses of HCW9218 45 days apart. Blood was drawn at various time points to assess immune cell subset frequencies. As expected, HCW9218 treatment mediated significant increases in the percentage of CD8^+^ T cells and NK cells in the blood which returned to baseline 4 weeks post‐treatment, with no evidence of tachyphylaxis at this interval (Figure 7f,g).

We could not demonstrate that the two‐dose treatment regimen provided a significant increase in the lifespan of naturally aged mice. HCW9218 contains human‐derived components and is known to be immunogenic inducing high titer of anti‐drug antibodies after two doses of treatment. This prohibited us from evaluating whether a more frequent dosing regimen could provide a survival advantage to naturally aged mice treated with HCW9218.

Overall, HCW9218 treatment was well tolerated by mice at dose levels significantly higher than the therapeutic level (3 mg/kg) we employed in this study, and no long‐term overt adverse effects of HCW9218 treatment were observed on the healthspan of naturally aged mice.

## 8 DISCUSSION

Cellular senescence, a state of irreversible arrested proliferation, plays a pivotal role in various physiological processes. However, the induction and accumulation of SNCs, caused by a variety of stressors promoting chronic inflammation through their accompanied SASP, are implicated in a wide spectrum of aging‐related diseases. Therefore, interest is rapidly growing in targeting SNCs to improve healthy aging and age‐related diseases (Gasek et al., 2021). Senolytic chemical drugs that selectively induce senescent cell death have been developed and are reportedly in early‐phase clinical studies with limited data on their efficacy (Hickson et al., 2019; Justice et al., 2019). Recently, chimeric antigen receptor T cell and antibody‐based targeting approaches to extracellular targets for immune‐mediated clearance of SNCs have been reported with promising results in animal models (Amor et al., 2020). However, significant challenges for these approaches include the highly heterogeneous transcriptome of SNCs due to a dependence on different stressors and cell types and temporally dynamic changes in gene expression profiles. As a result, so far there is no unique cell‐surface marker identified for targeting SNCs. On the contrary, there is evidence showing that cellular senescence can be controlled by immune response leading to senescent cell elimination (Papismadov & Krizhanovsky, 2021). SNCs upregulate NKG2D ligands on their surface, which are recognized by NK cells and NKT cells for cell‐mediated killing (Brighton et al., 2017; Kale et al., 2020).

In a recent study, we showed that HCW9218 promotes NK‐cell‐mediated clearance of chemotherapy‐induced cancer and normal tissue SNCs (Chaturvedi et al., 2022). Herein, we clearly demonstrate that subcutaneously administered HCW9218 potently activates and promotes the fitness of NK and CD8^+^ T cells in metabolically dysfunctional and naturally aged mice. HCW9218‐mediated immune activation correlated with a reduction of senescent pancreatic β cells, SASP, and the aging index, as well as with improvements in blood glucose and insulin resistance in T2D *db/db* mice. These findings are consistent with a recent study demonstrating that the removal of senescent β cells resulted in improved T2D disease outcomes in mice (Aguayo‐Mazzucato et al., 2019). Remarkably, RNA‐Seq analysis also showed that HCW9218 treatment broadly affected the transcriptome of liver genes related to gluconeogenesis, lipid metabolism, cellular senescence, and inflammation which may contribute to the overall improvement in health as observed in HCW9218 treated diabetic *db/db* mice. We hypothesize that this global effect in the liver is the result of removal of p21^+^ SNCs mediated by HCW9218‐activated immune cells. p21, in addition to its function in maintaining the cell cycle‐arrest in SNCs, is known to have a prominent role in establishing SASP (Sturmlechner et al., 2021). Thus, the reduction of SASP by removing p21^+^ SNCs is expected to lessen the systemically deleterious paracrine effects of SASP in islet β cell dysfunction. Interestingly, we observed that the expression of *Tert* was upregulated by HCW9218 treatment in the aged mice. TERT is implicated in telomere attrition causing replicative senescence in aged mice. It should also be noted that, unlike in TIS cells, we did not observe downregulation of *Cdkn2a* expression, another major pathway leading to cellular senescence, with HCW9218 treatment in naturally aged mice. In human fibroblasts, p21 was decreased after senescence establishment and p16 is upregulated for senescent‐cell‐cycle arrest maintenance (Stein et al., 1999). Thus, it seems puzzling why we observed p21^+^ but not p16^+^‐cell reduction 6 months after two doses of HCW9218 treatment in naturally aged mice. We hypothesize that this may be the result of a difference in the magnitude or duration of p21^+^ versus p16^+^‐cell clearance induced by HCW9218 treatment. Six months after the two‐dose regimen of HCW9218, the levels of p16^+^ cells but not the p21^+^ cells accumulated back to the pre‐HCW9218 treatment level. It has been shown that genetic ablation of p16^+^ cells in a transgenic mouse model could increase the lifespan of the mice versus their p16 wild‐type counterparts (Baker et al., 2016). In our study, the two‐dose regimen of HCW9218 treatment did not increase the lifespan of naturally aged mice compared to untreated mice, although HCW9218 treatment provided major benefits in the improvement of physical strength to naturally aged mice without compromising their healthspan. We are initiating additional studies to examine whether a prolonged multiple‐dose regimen of HCW9218 could reduce p16^+^ cells in the organs and prolong the lifespan of naturally aged mice. Further studies also are needed to explore whether cellular senescence induction in naturally aged mice is similar to TGF‐β1 induced cellular senescence in glomerular endothelial cells via CDKN2A (p16) translocation from cytosol to nucleus (Ueda et al., 2021). A recent study showed that clearance of p21‐ but not p16‐positive senescent cells prevented radiation‐induced osteoporosis and increases marrow adiposity (Chandra et al., 2022). Therefore, the relevance of p21 and p16 in cellular senescence induction in naturally aged mice should be thoroughly evaluated.

In naturally aged mice, activation of the immune system and neutralization of TGF‐β by HCW9218 administration correlated with a durable reduction of SNCs and SASP in peripheral organs. Group 1 ILCs are tissue‐resident immune cells comprised of both NK cells and ILC‐1s and are particularly enriched in the liver. IL‐15‐activated NK and ILCs exhibit cell‐mediated cytotoxicity against target cells through granule exocytosis (Chaturvedi et al., 2022; Nixon et al., 2022). In this study, we showed that HCW9218 potently stimulated, promoted expansion, and enhanced the cytotoxicity of group 1 ILCs in the livers of aged mice. It is known that these cells can positively and negatively influence adaptive immune responses (Bal et al., 2020). Thus, while we presume the granzyme‐mediated cytotoxicity or the secreted effector molecules (e.g., IFNγ) of HCW9218‐activated group‐1 ILCs are responsible for the SNC‐reducing activities, ILCs could also play an anti‐inflammatory role in SASP. For example, it has been recently shown that group‐1 ILCs regulate T cell‐mediated liver immunopathology induced in hepatitis by controlling local IL‐2 availability (Fumagalli et al., 2022).

Similar to diabetic mice, we observed effects of HCW9218 treatment on the liver transcriptome of aged mice. Besides the traditionally considered SASP proinflammatory genes, we observed a significant reduction of *Lcn2*, *S100a8*, *S100a9*, *S100a11*, *Mt1*, and *Mt2* expression by HCW9218 treatment. LCN2 is released by various cell types and acts as a master mediator of intestinal and metabolic inflammation. In animal models of metabolic inflammation, T2DM, or nonalcoholic steatohepatitis, increased LCN2 expression promotes inflammation through the recruitment of inflammatory cells and induction of proinflammatory cytokines (Moschen et al., 2017). Proinflammatory cytokines S100A8/S100A9/S100A11 are calcium‐binding proteins that are upregulated in human cancer. They are involved in tumor growth, metastasis, angiogenesis, immune evasion, metabolic diseases, neurological diseases, and vascular calcification. S100A8/A9 released by cancer cells and tissues interacts with Toll‐like receptor 4 and Receptor for Advanced Glycation End (RAGE) products to direct chemotaxis of myeloid cells and induces proinflammatory responses and/or immune suppressions by activating JAK–STAT, NF‐κB, and MAPK pathways. Recently, it has also been shown that S100A8/S100A9 and S100A11 are recruited to numerous promoters and enhancers to act as transcriptional coactivation molecules. These signal transduction pathways activated by S100A8/S100A9/S100A11 are the key pathways for SASP induction and are the targets for senomorphic drug development (Zhang et al., 2022). MT1 and MT2 are involved in regulating steady‐state concentrations of various metals, with a role in alleviating heavy metal poisoning and protecting the body against oxidative stress, inflammation, and other cell damage caused by stress reactions. The significant downregulation of *Mt1* and *Mt2* transcripts by HCW9218 treatment may reflect the re‐establishment of immune homeostasis by removing stressors such as the proinflammatory cytokines caused by the accumulation of SNCs and SASP.

In addition to downregulation of senescence and SASP related genes, HCW9218 treatment unexpectedly reduced transcription of *Bmal* and *Clock*, and increased transcription of *Nr1d1/Nr1d2*, *Dbp*, *Per1*, *Per2*, *Cry1*, and *Cry2* genes in the liver of aged mice. These genes control the circadian rhythm, and their imbalance or mis‐regulation is implicated in many diseases, including Alzheimer's disease (Acosta‐Rodriguez et al., 2021; Clark et al., 2022; Nassan & Videnovic, 2022). In addition to their regulation of circadian rhythms, these clock gene products are known to have a variety of homeostatic functions. For example, NR1D1 was shown to modulate synovial inflammation and bone destruction in rheumatoid arthritis (Liu et al., 2020). DBP and TEF play a pivotal role in xenobiotic detoxication, and PER3 functions as a potent mediator of cell fate by altering the transcriptional activity of PPARγ (Costa et al., 2011; Gachon et al., 2006). Interestingly, TGF‐β acts as zeitgeber for peripheral clocks and has been recently identified as a peripheral coupler that mediates paracrine‐phase adjustment of molecular clocks through transcriptional regulation of core‐clock genes *in vitro* (Finger et al., 2021; Kon et al., 2008). Neutralization of TGF‐β by HCW9218 treatment, therefore, could lead to the transcriptional changes of the core‐clock genes in the liver of aged mice as shown in this study. In addition, it has recently been shown that BMAL1, encoded by *Arntl1*, stabilizes heterochromatin and prevents activation of the LINE1‐cGAS‐STING pathway which is known to play a key role in cellular senescence and SASP (Liang et al., 2022). A deficiency of BMAL1 results in accelerated aging phenotype in both human and non‐human primate mesenchymal progenitor cells. Given the importance of circadian rhythm for aging, longevity, and metabolic homeostasis (Acosta‐Rodriguez et al., 2021; de Assis & Oster, 2021; Mukherji et al., 2020), the ability of HCW9218 treatment to restore the circadian rhythm should be further investigated as a novel mechanism for mitigating age‐related physical decline and chronic diseases.

TGF‐β1, secreted as part of SASP, can induce or accelerate, and maintain the senescent phenotype in various cell types including fibroblasts, bronchial epithelial cells, and cancers in an autocrine/paracrine manner (Debacq‐Chainiaux et al., 2005; Minagawa et al., 2011; Senturk et al., 2010). For example, TGF‐β‐1 induces vascular smooth muscle cell (VSMC) senescence through reactive oxygen species (ROS)‐stimulated activation of NF‐κB signaling and expression of SASP, including plasminogen activator inhibitor type‐1 (*PAI‐1*, *SERPINE1*). PAI‐1 is not only a biomarker of cellular senescence but also is necessary and sufficient for replicative senescence downstream of p53 and is a key inducer of the senescence program (Hiebert et al., 2018; Kortlever et al., 2006). There is evidence for a PAI‐1/TGF‐β1‐positive feed‐forward mechanism that supports tissue levels of TGF‐β1 during the emergence of the senescent phenotype stimulating expression of PAI‐1 that, in turn, reinforces continued TGF‐β1 synthesis (Seo et al., 2009), thereby promoting the maintenance, and perhaps expansion, of the senescent VSMC population. Results in this study suggest that interference of TGF‐β signaling by the TGF‐βRII component of HCW9218 can directly lower the expression of *Cdkn1*a which has been shown to alter the transcriptional regulatory properties of retinoblastoma protein to inhibit genes required for cell‐cycle progression while activating a large collection of genes implicated in diverse biological functions, including SASP (Sturmlechner et al., 2021). Thus, our findings support the concept of using TGFβRII as a senomorphic agent. This is also consistent with the results of studies showing that the canonical TGF‐β signaling Smad3/4 complex interacts with FOXO subfamily proteins, and Smad3 interacts with Sp1, a Zn finger transcription factor, to bind to the distal and proximal regions of *Cdkn1a*, respectively, activating p21 expression (Pardali et al., 2000; Seoane et al., 2004). However, the use of TGFβRII alone (HCW9228), as shown in RNA‐seq analysis, may have a more limited impact on SNCs and SASP (Figure S3C) when compared to HCW9218 treatment.

SNCs play an important role in a variety of biological functions. Therefore, one concern regarding senolytic and senomorphic drugs is that their activity could reduce the potential benefits of SNCs and SASP. In a dose‐ranging GLP toxicology study in mice, two‐dose subcutaneous administration of HCW9218 did not result in any overt adverse events even at high dose levels. In a long‐term multidose study in 72‐week‐old mice, we also did not observe any adverse differences in the healthspan of HCW9218‐treated versus un‐treated animals more than 6 months after receiving treatment, although the fasting glucose level was significantly lowered in the treatment group. This is likely the result of the combined effect of HCW9218 treatment to modulate gluconeogenesis and lipid‐metabolism genes and to lower *Angptl4* expression. Hepatocyte‐specific suppression of *Angptl4* expression was recently shown to improve obesity‐associated diabetes and mitigate atherosclerosis in mice (Singh et al., 2021).

We have previously shown that IL‐15/IL‐15Rα agonists are potent activators of immune cells, particularly NK cells (Rhode et al., 2016). Therefore, IL‐15 agonists are expected to support immune‐cell‐mediated clearance of senescent cells if these agonists exhibit similar pharmacokinetic profiles as HCW9218. This is consistent with our observations in this study that some of the beneficial effects of HCW9218 are ameliorated when mice were treated with HCW9228 which lacks IL‐15 activities. However, since TGF‐β is a potent immunosuppressive cytokine and the master regulator of SASP, HCW9218 with combined TGF‐β neutralizing and immune‐cell activation capabilities could be a more effective immunotherapeutic than IL‐15 agonists for senescent‐cell removal and SASP alleviation.

In conclusion, we have shown that a non‐targeted immunotherapeutic with combined IL‐15 activity and TGF‐β neutralization promotes the activation and durable fitness of immune cells and could represent a new class of safe and effective SNC‐reducing and senomorphic drugs for anti‐inflammaging uses. HCW9218 is currently in two clinical trials against chemotherapy‐resistant solid tumors and pancreatic cancer (ClinicalTrials.gov: NCT05304936 and NCT05322408). The results of these studies will provide guidance to a safe regimen for future development of HCW9218 to treat other age‐related pathologies.

## AUTHOR CONTRIBUTIONS

HCW, NS, PC, XZ, PRR, and TAF involved in conceptualization. HCW, NS, PC, MD, VG, XZ, TAF, CJ, MBE, and JE involved in methodology. MD, PC, NS, VG, JE, XZ, BL, MBE, MF, LM, PW, CCC, JF, JT, TS, KH, GL, LY, CE, CS, CJ, LK, CG, NE, LK, MW, BF, and ZW involved in investigation. MD, PC, NS, VG, XZ, MBE, JE, CJ, BL, LL, and LK involved in visualization. HCW and TAF involved in funding acquisition. HCW and NS involved in project administration. HCW, TAF, NS, and PC involved in supervision. HCW, NS, PC, and XZ involved in writing—original draft. HCW, PRR, NS, PC, XZ, MD, VG, TAF, MBE, CJ, JE, BL, GL, and RD involved in writing—review and editing.

## FUNDING INFORMATION

This study was supported by HCW Biologics Inc. Some of the research reported in this publication were also supported by grants from the National Institutes of Health (NIH) National Cancer Institute (P50CA171963 to TAF and MMB‐E; R01CA205239 to TAF; P30CA91842 to TAF and Siteman Cancer Center Immunomonitoring Lab, and K12CA1667540 to MMB‐E), NIH National Heart, Lung, and Blood Institute (T32HL007088 to JAF and PW), NIH National Institute of General Medical Sciences (T32GM139799 to JAF), NIH National Institute of Allergy and Infectious Diseases (F30AI161318 to CCC) and the Siteman Cancer Center Investment Program to TAF.

## CONFLICT OF INTEREST STATEMENT

HCW9218 is protected under US Patent No. 11,518, 972 and other pending patent applications. Unrelated to this work, MMB‐E and TAF are inventors on patent/patent applications (15/983,275; 62/963,971, and PCT/US2019/060005) licensed to Wugen, Inc. and held/submitted by Washington University that covers aspects of ML NK cell biology. This results in potential royalties to MMB‐E, TAF, and Washington University from Wugen, Inc. MMB‐E, TAF consults for and has equity in Wugen Inc. Unrelated to this work, JAF is an inventor on patent/patent application (WO 2019/152387, US 63/018,108) licensed to Kiadis, Inc. and held/submitted by Nationwide Children's Hospital on TGF‐β resistant, expanded NK cells. Unrelated to this work, JAF has a monoclonal antibody unrelated to the present work licensed to EMD Millipore. Unrelated to this work, CCC reports equity in Pionyr Immunotherapeutics. Unrelated to this work, TAF consults for Affimed (SAB) and advises (equity interest) Indapta and OrcaBio.

## Supporting information


AppendixS1

AppendixS2

AppendixS3

## Data Availability

The proprietary materials are available in limited quantities at a reasonable cost to the scientific community for non‐commercial purposes, upon timely request under a material transfer agreement through HCW Biologics Inc. RNA‐seq data for Figures, 2L‐P were deposited to SRA and can be accessed with Accession# PRJNA927323. Other data for RNA‐Seq and single‐cell RNA‐Seq are submitted as in supplementary material.
