# Supplementary material for: Immunotherapeutic approach to reduce senescent cells and alleviate senescence‐associated secretory phenotype in mice
Source: Aging Cell. 2023 Mar 26;22(5):e13806. doi: 10.1111/acel.13806 (PMC10186597; doi:10.1111/acel.13806)
Supplement: Supplementary file 1 — AppendixS1 [file ACEL-22-e13806-s002.pdf]

Supplementary Figures

Fig. S1. Metabolic activities of splenocytes of *db/db* mice. (A-D) Representative data for increase in metabolic parameters, (A) Glycolytic parameters, (B) Mitochondrial respiration, from splenocytes of db/db mouse stimulated *in vivo* with one dose of HCW9218 and HCW9228 and analyzed by Seahorse XFe bioanalyzer. (A-B) Represents data from day 2 and (C-D) represents data from day 4 post treatment. *p* values were determined by ordinary one-way ANOVA with Tukey's multiple comparisons test.

A.

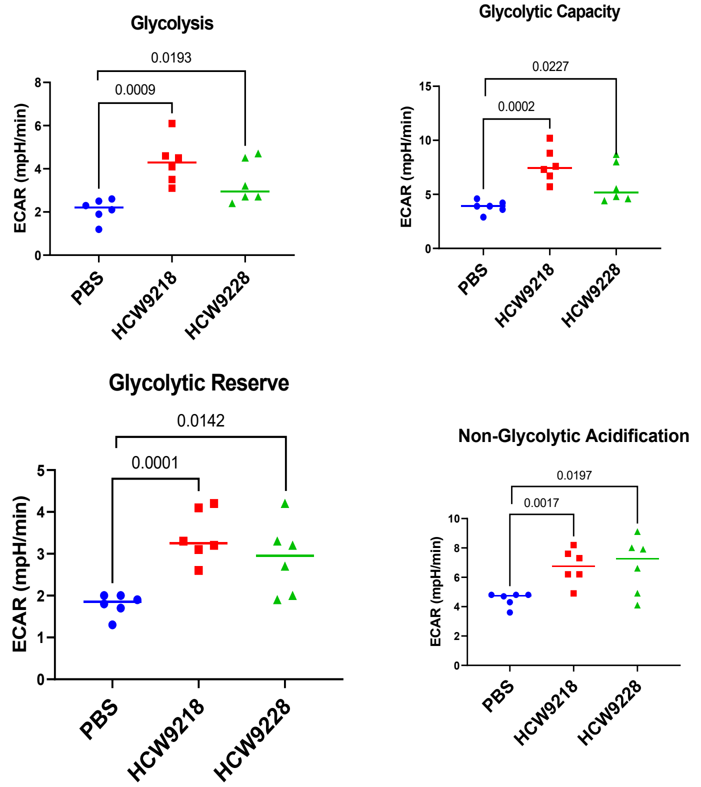

B.

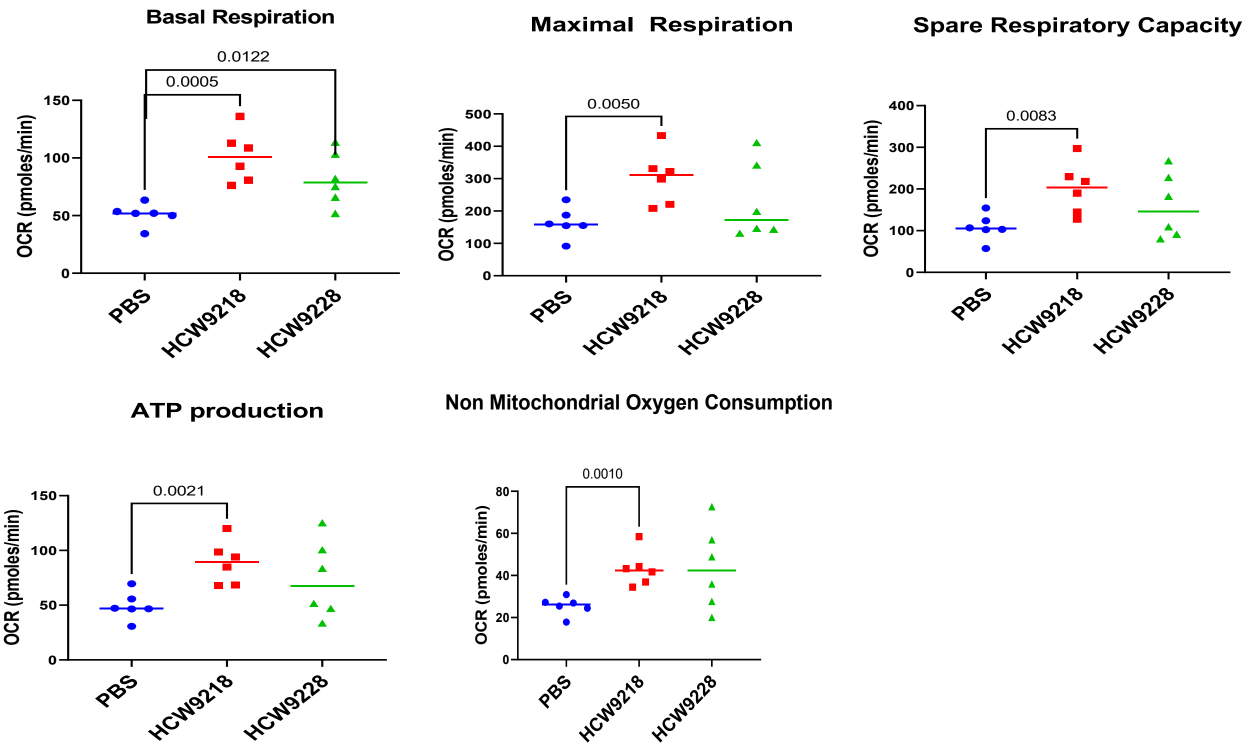

C.

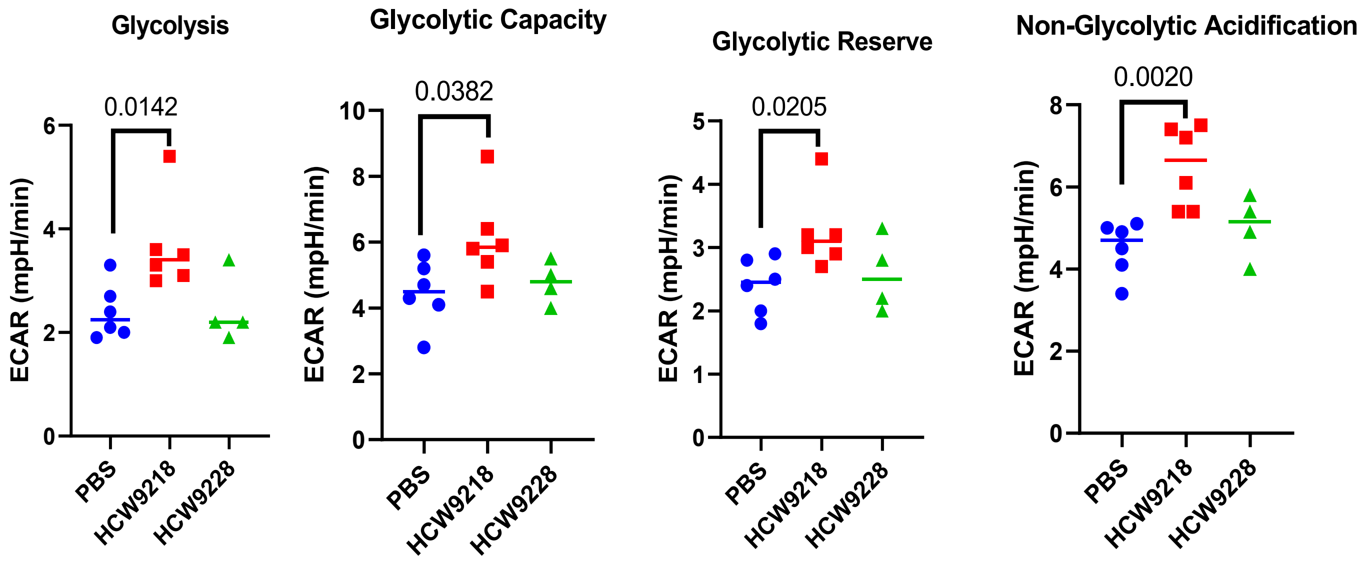
Glycolysis
0.0142
ECAR (mpH/min)
Glycolytic Capacity
0.0382
ECAR (mpH/min)
Glycolytic Reserve
0.0205
ECAR (mpH/min)
Non-Glycolytic Acidification
0.0020
ECAR (mpH/min)
PBS
HCW9218
HCW9228

D.

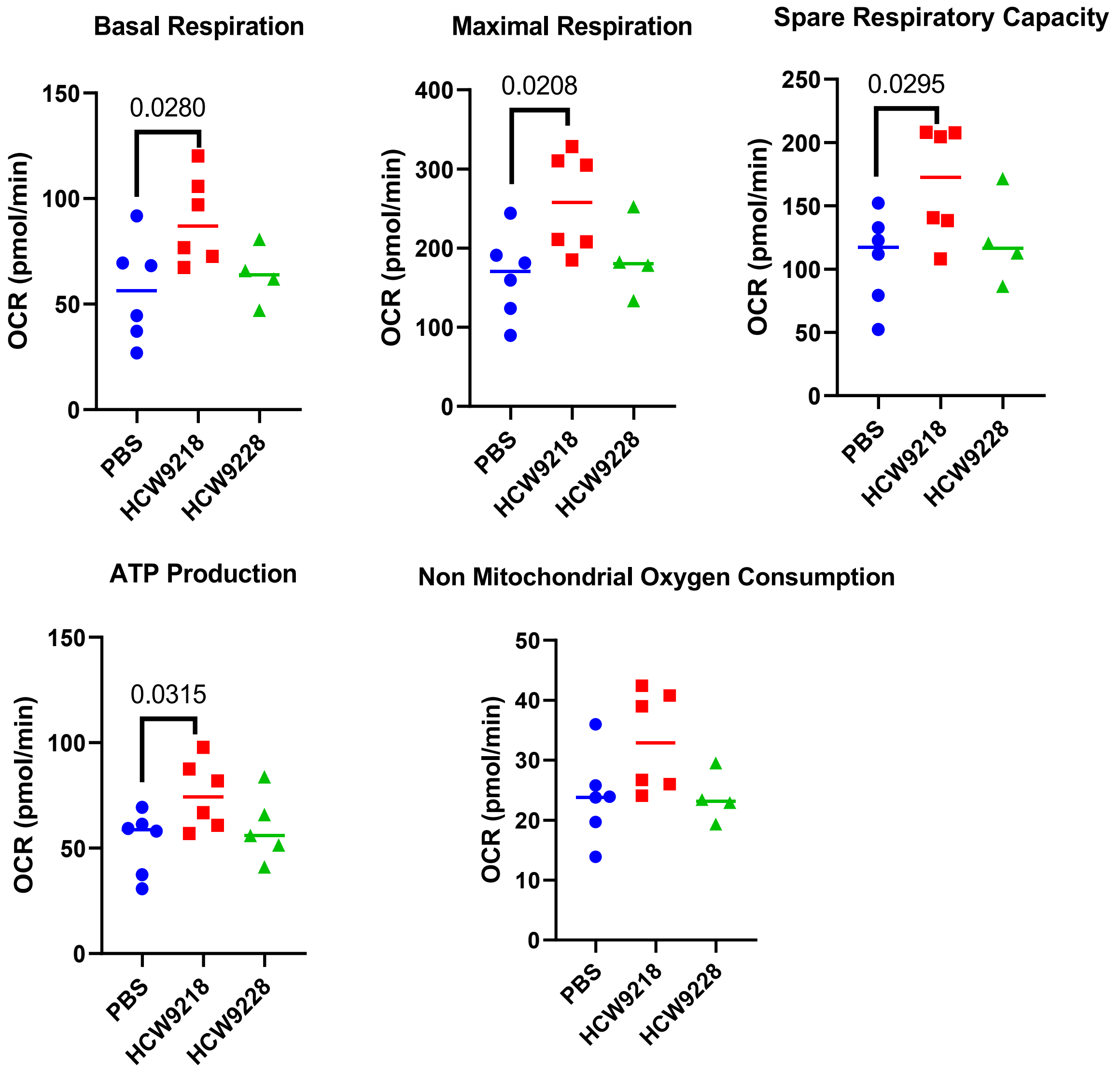
Basal Respiration
0.0280
OCR (pmol/min)
Maximal Respiration
0.0208
OCR (pmol/min)
Spare Respiratory Capacity
0.0295
OCR (pmol/min)
ATP Production
0.0315
OCR (pmol/min)
Non Mitochondrial Oxygen Consumption
OCR (pmol/min)
PBS
HCW9218
HCW9228

Table. S1. Summary table of liver RNA Seq data showing 33 genes.

Group	Regulation	ID	log2FC	pvalue	padj	Gene
Glucose regulation	Down	ENSMUSG00000012705	-2.12306	5.09E-05	0.021507	Retn
	Down	ENSMUSG00000018566	-1.80962	7.35E-06	0.004942	Slc2a4
	Down	ENSMUSG00000019577	-1.34542	3.76E-10	1.08E-06	Pdk4
	Down	ENSMUSG00000041653	-1.32796	1.08E-06	0.000967	Pnpla3
	Down	ENSMUSG00000015312	-1.04635	8.53E-05	0.032995	Gadd45b
	Down	ENSMUSG00000029167	-1.0363	2.79E-06	0.002228	Ppargc1a
	Down	ENSMUSG00000027452	-2.16447	0.000148	0.046106	Acss1
	Down	ENSMUSG00000020264	-2.53609	7.57E-06	0.004942	Slc36a2
Senenscent cell regulation	Down	ENSMUSG00000007655	-1.61384	0.000104	0.034636	Cav1
	Down	ENSMUSG00000037419	-1.22862	2.82E-05	0.014052	Endod1
	Down	ENSMUSG00000019577	-1.34542	3.76E-10	1.08E-06	Pdk4
	Down	ENSMUSG00000015312	-1.04635	8.53E-05	0.032995	Gadd45b
Inflammation regulation	Up	ENSMUSG00000032578	1.447471	6.18E-08	8.88E-05	Cish
	Down	ENSMUSG00000030017	-3.11365	7.11E-05	0.029176	Reg3g
	Down	ENSMUSG00000076613	-2.82776	3.06E-18	4.40E-14	Ighg2b
	Down	ENSMUSG00000064057	-2.76399	9.05E-05	0.032995	Scgb3a1
	Down	ENSMUSG00000022491	-2.63969	3.88E-07	0.000507	Glycam1
	Down	ENSMUSG00000076612	-2.59181	2.67E-08	4.56E-05	Ighg2c
	Down	ENSMUSG00000076609	-2.46752	1.39E-15	9.97E-12	Igkc
	Down	ENSMUSG00000024673	-2.42377	8.34E-05	0.032995	Ms4a1
	Down	ENSMUSG00000067149	-2.06336	2.86E-08	4.56E-05	Jchain
	Down	ENSMUSG00000076617	-2.03893	6.19E-11	2.22E-07	Ighm
	Down	ENSMUSG00000079017	-1.91748	8.57E-07	0.000856	Ifi27l2a
	Down	ENSMUSG00000044337	-1.68889	4.63E-06	0.003327	Ackr3
	Down	ENSMUSG00000018819	-1.46106	9.06E-05	0.032995	Lsp1
	Down	ENSMUSG00000038400	-1.11498	4.45E-06	0.003327	Pmepa1
	Down	ENSMUSG00000018339	-1.04274	3.87E-05	0.017861	Gpx3
Vascular regulation	Down	ENSMUSG00000055775	-5.78706	2.84E-05	0.014052	Myh8
	Down	ENSMUSG00000041616	-5.67018	6.68E-07	0.000738	Nppa
	Down	ENSMUSG00000007877	-3.45345	2.00E-06	0.001691	Tcap
	Down	ENSMUSG00000040752	-2.32434	1.71E-14	8.20E-11	Myh6
	Down	ENSMUSG00000068614	-2.11515	2.07E-05	0.011003	Actc1
	Down	ENSMUSG00000035783	-1.5518	3.98E-05	0.017861	Acta2
	Down	ENSMUSG00000028464	-1.28337	6.32E-07	0.000738	Tpm2
	Down	ENSMUSG00000044041	-6.76486	6.73E-09	1.38E-05	Krt13

Fig. S2. HCW9218 enhanced immune-mediated biological activities in naturally-aged mouse (A-B). Representative data by flow cytometry showing increase in immune cell surface makers compared to controls at day 4 after treatment with HCW9218 in blood (A) and spleen (B) of young and aged mice. (C-D). Immune cells proliferation marker Ki67 in NK cells in blood (C) and spleen (D) by flow cytometry at day 4 of young and aged mice. (E-G) Representative flow cytometry data showing increase in immune cell surface makers on liver immune cells compared to controls at day 4 after treatment with HCW9218 in young and aged mice. Individual value plot show the means of five mice per group from two independent experiments. *p* values were determined by Student's *t* tests for two group comparison and *p* values were determined by ordinary one-way ANOVA with Tukey's multiple comparisons test where there are more than two groups.

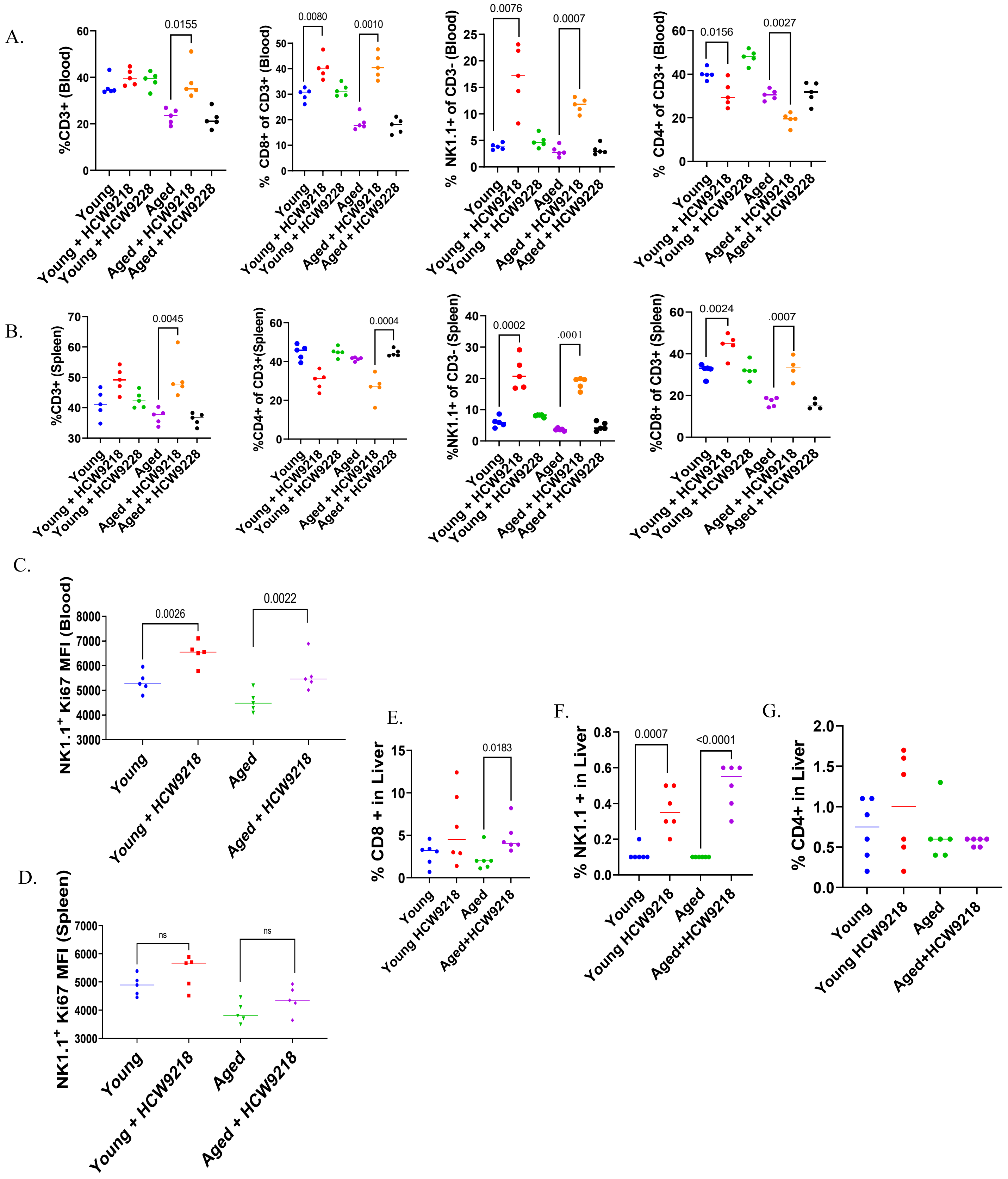

Fig. S2. Cont. HCW9218 stimulates immune cell activity in liver and spleen of naturally-aged mice. (J-K) Summary data from 2 independent experiments, with 6 mice/group demonstrating the increased in frequency of total CD4$^+$ T cells in liver at day 4 and day 10 after treatment with HCW9218 compared to control treatment and in spleen at day 4 and 10. Individual value plot show the means of six mice per group from two independent experiments. *p* values were determined by ordinary one-way ANOVA with Tukey's multiple comparisons test where there are more than two groups.

J.

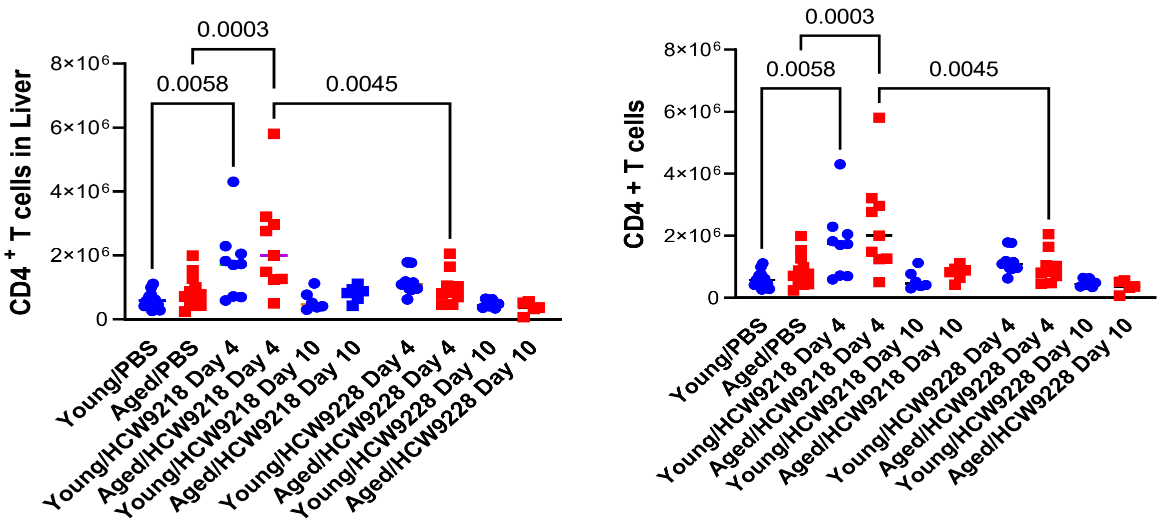

K.

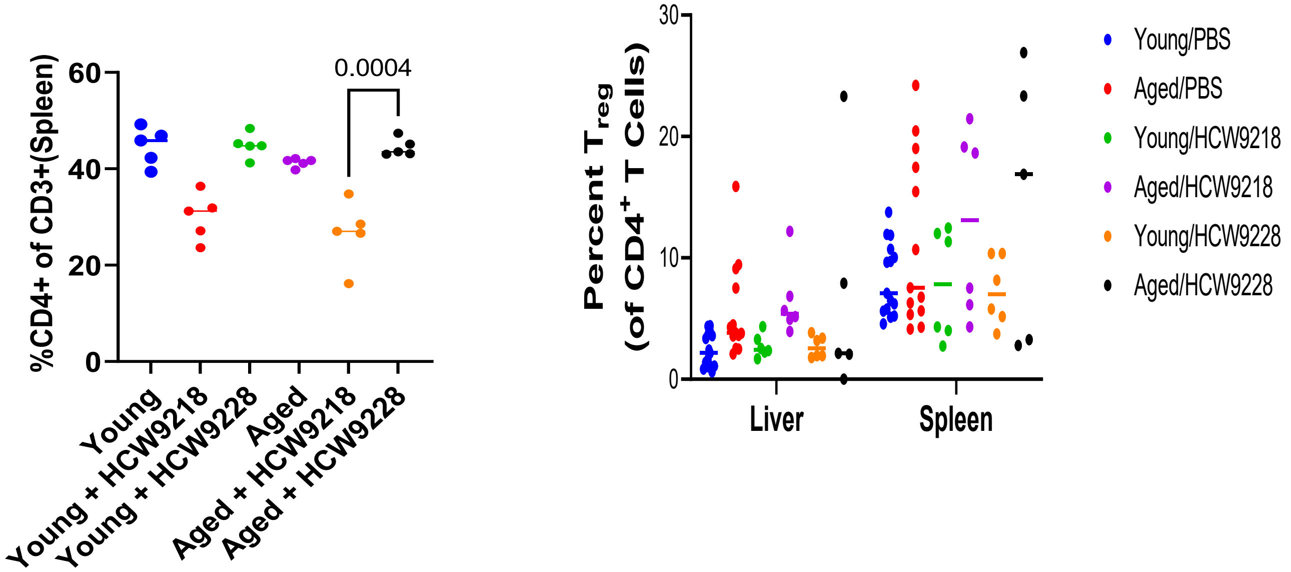

Fig. S3. Metabolic parameters of splenocytes following HCW9218 treatment in young and naturally-aged mice. (A-B) Representative data for metabolic parameters from splenocytes from young and naturally aged mouse stimulated *in vivo* with one dose of HCW9218 and HCW9228 by Seahorse XFe bioanalyzer compared to control at day 4 (A-B). *p* values were determined by ordinary one-way ANOVA with Tukey's multiple comparisons test.

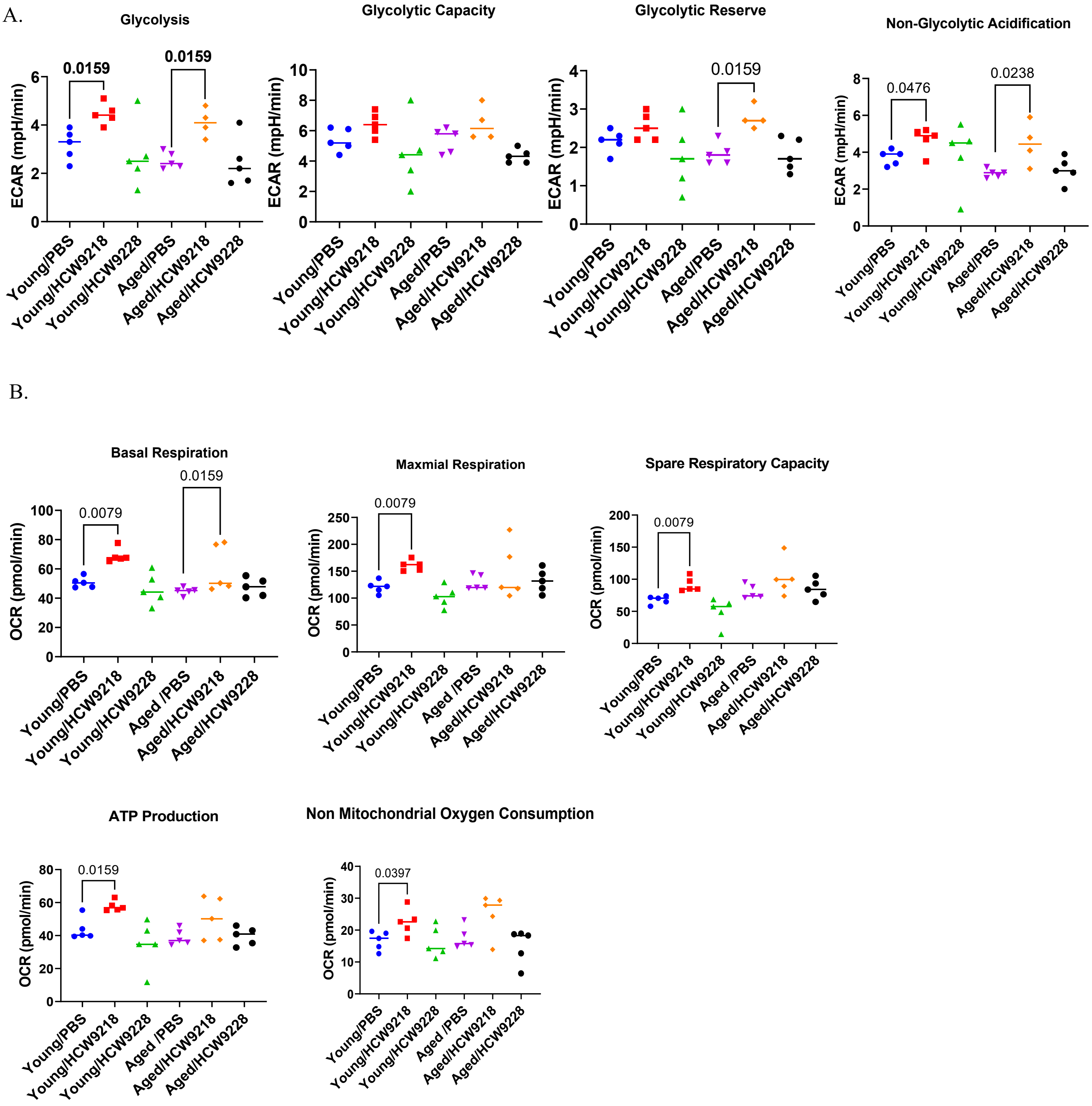

Fig. S4. HCW9218 stimulates immune cell activity and metabolic functions and reduces inflammation (SASP) and cellular senescence markers of naturally-aged mice. (A) Heat maps of the differentially expressed immune pathway associated genes in liver after treatment with HCW9218 compared to control treatment (adjusted *p* value <0.05).

A.

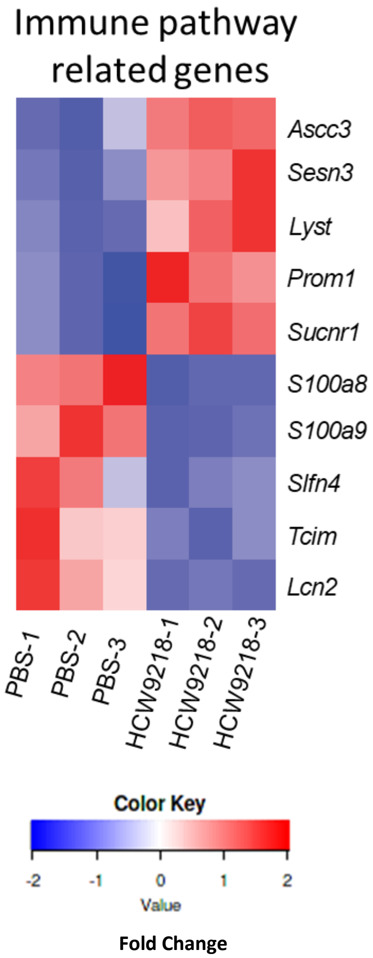

Fig. S5. Metabolic activities of splenocytes following HCW9218 treatment in naturally-aged mice. (A-B) Representative data of metabolic parameters from splenocytes from aged mouse stimulated *in vivo* with two dose of HCW9218 and HCW9228 by Seahorse XFe bioanalyzer compared to control at day 90 (A-B). *p* values were determined by ordinary one-way ANOVA with Tukey's multiple comparisons test where there are more than two groups.

A.

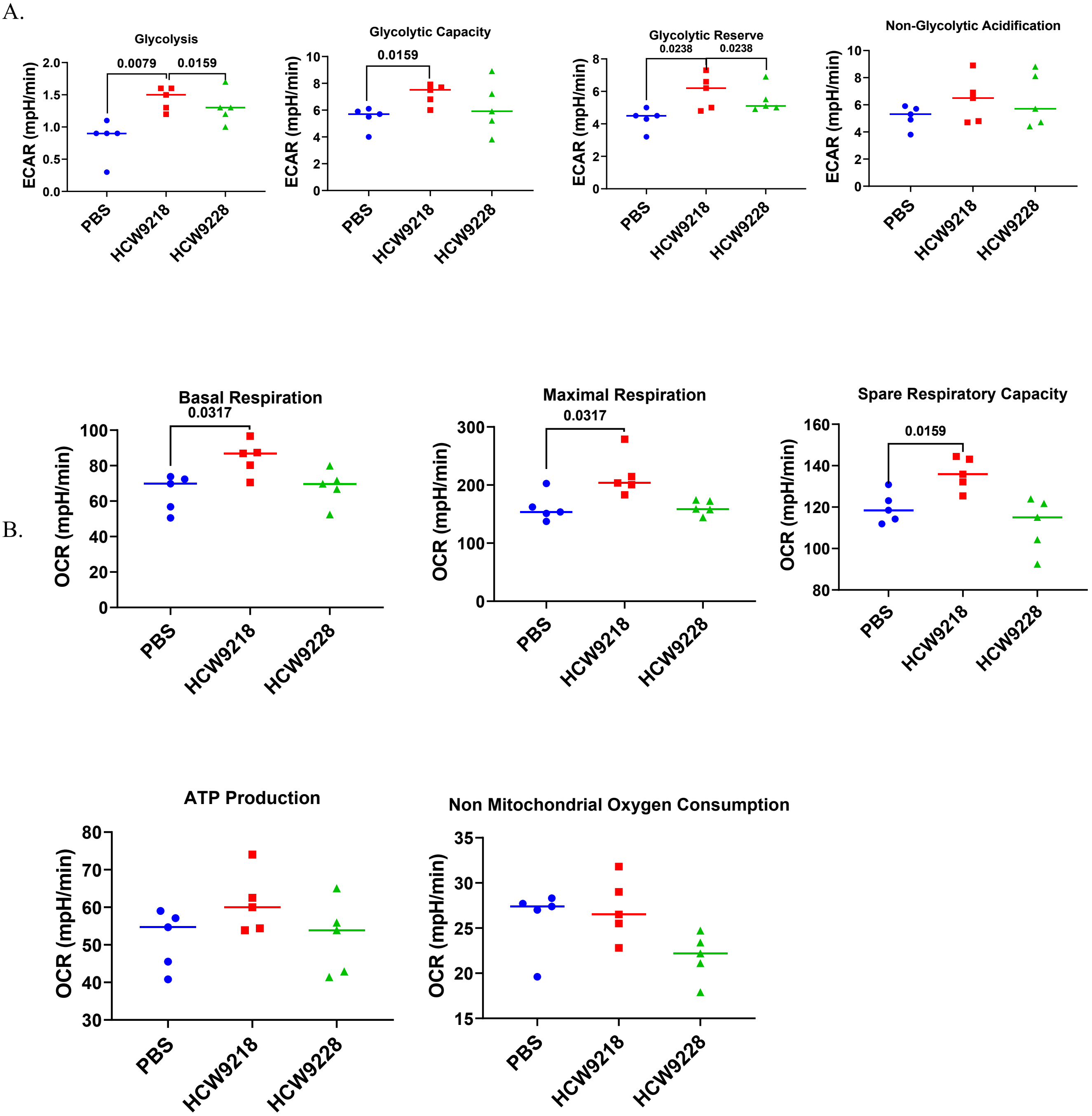

Table S2. Table for differentially expressed genes in liver associated with different pathway analyzed by KEGG pathway after treatment with HCW9218 compared to control treatment at day 120.

Pathway	*p* value	No. of genes	Genes
Inflammatory response	4.26E-07	26	*Clec10a Ccl24 Tnfaip8l2 Fcgr1 Ccl6 Ccl4 Ly86 Trem2 Casp1 Cxcl9 Cd300a Ccl2 Tlr7 Tlr1 Tlr6 Pla2g4a Sphk1 Ccr5 Rgs1 SA100A10 S100a1 S100a4 S100a16 S100a6 S100g Il18*
Response to stress	0.000378959	30	*Timp1 Cyba Cd40 Wfdc15b ENSMUSG00000018211 Gpx8 Cd200r1 Cd86 ENSMUSG00000022901 Clec4n Aif1 Casp12 Col3a1 Ube2t Stap1 Klrd1 Rad51ap1 Tyrobp Pycard Gins2 Casp4 Gbp8 Ubd Cela2a Gm4841 Lyz2 Wfdc17 Lilra5 Bcl2a1b Bcl2a1a*
Regulation of MAPK cascade	1.43E-06	15	*Ccl24 Ace2 Ccl6 Ccl4 Dok2 Angpt1 Trem2 Fgd2 Rgs2 Ptprc Gilpr2 Cd300a Ccl2 Tlr6 Sphk1*
Interleukin-6 production	0.000529835	6	*Cyba Cd200r1 Aif1 Tyrobp Pycard Lilra5*
Non-alcoholic fatty liver disease	0.002941686	17	*Sdhd Ndufb2 Cox6c Ndufa1 Cox7c Uqcr11 Eif2s1 Ndufs4 Ndufb9 Ndufb3 Ndufs5 Ndufa4 Cox4i1 Cox7a2 Uqcrq Uqcr10 Ndufb6*
Cellular senescence	0.001159093	13	*Cdk1 Gadd45g Rbl1 Chek2 Calml4 Gadd45a Cdkn2b Cxcl9 Rn7sk Cxcl1 Cox7c Casp4 Mmp12*
Cytokine-cytokine receptor interaction	0.000410128	16	*Ccl24 Ccl6 Ccl4 Cxcl9 Gdf3 Ccl2 Il7 Ccr5 Il18 Il15 Ccl7 Ccl8 Cxcl1 Cxcl10 Cxcl11 Ccl27*
p53 pathway	0.007080325	4	*Cdk1 Rrm2 Chek2 Gadd45a*
Metabolic pathways	0.006830825	58	*Srr Cyp2c29 Itpkc Gys1 Gstm3 Fpgs Hyal2 Ehmt2 St3gal1 Prune1 Pgs1 B4galt5 Plod1 Entpd5 Pde4d Acox2 Naga Lpin2 Xdh Gpam Shmt2 Hadha Mgat4a Atic Uck2 Inpp5e Itpka Impa1 Papss1 Plpp3 Inpp5b Mmab Fah Cdipt Got2 Tecr Dpagt1 Cyp2c38 Pomt2 Agpat1 Aldh1b1 Hykk Mgat5 Pafah2 Cox15 Maob Pigs Fkrp Sgms2 Sphk2 Itpk1 Cds2 Mocs1 mt-Atp8 mt-Atp6 Dgkd G6pc Tmem189*
Immune system process	3.46E-07	29	*Impdh1 Il7r Pou2f2 Dock2 Irf4 Hcls1 Csf1r Marco Apbb1ip Rassf2 Samhd1 Oasl2 Dtx1 Planp Cd37 Elf4 Msn Il2rg Smpd3 Oas3 Rac2 Card11 Tgfb2 Sh3kbp1 Cmklr1 Spn Nfam1 Il2rb Runx3*
Regulation of immune system process	4.03E-07	21	*Il7r Irf4 Hcls1 Csf1r Rassf2 Samhd1 Dtx1 Planp Cd37 Msn Il2rg Smpd3 Oas3 Rac2 Card11 Tgfb2 Sh3kbp1 Cmklr1 Spn Nfam1 Runx3*
T cell activation	9.97E-06	12	*Il7r Dock2 Irf4 Apbb1ip Dtx1 Elf4 Msn Il2rg Rac2 Card11 Spn Runx3*
Apoptotic signaling pathway	8.25E-05	20	*Pias4 Erbb3 Stk4 Nf1 Slc9a3r1 Tert Grina Gsk3b Tmbim6 Asah2 Rhot2 Zdhhc3 Ptpn1 Ube4b Sh3rf1 Gnai2 Wfs1 Foxo3 Cebpb Stk24*

Fig. S6. Heat maps of the differentially expressed Senescence, Inflammation and Circadian Rhythm associated genes in liver after treatment with HCW9228 compared to control treatment (adjusted *p* value <0.05).

A.

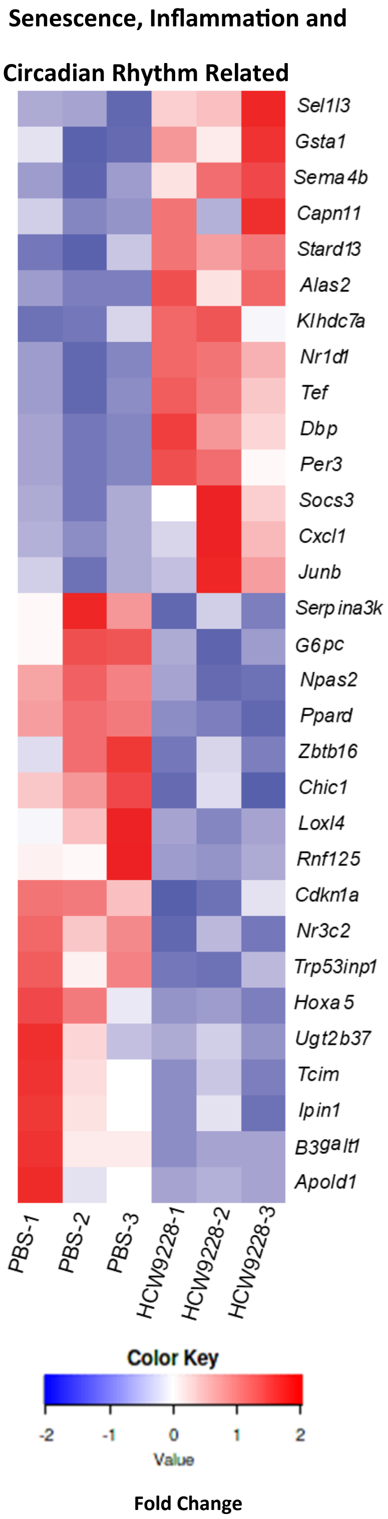

Fig. S6. Cont. (B) Heat maps of the differentially expressed Senescence, Inflammation and Circadian Rhythm associated genes associated genes in liver after treatment with HCW9218 compared to HCW9228 treatment after 120 days (adjusted p value <0.05).

B.

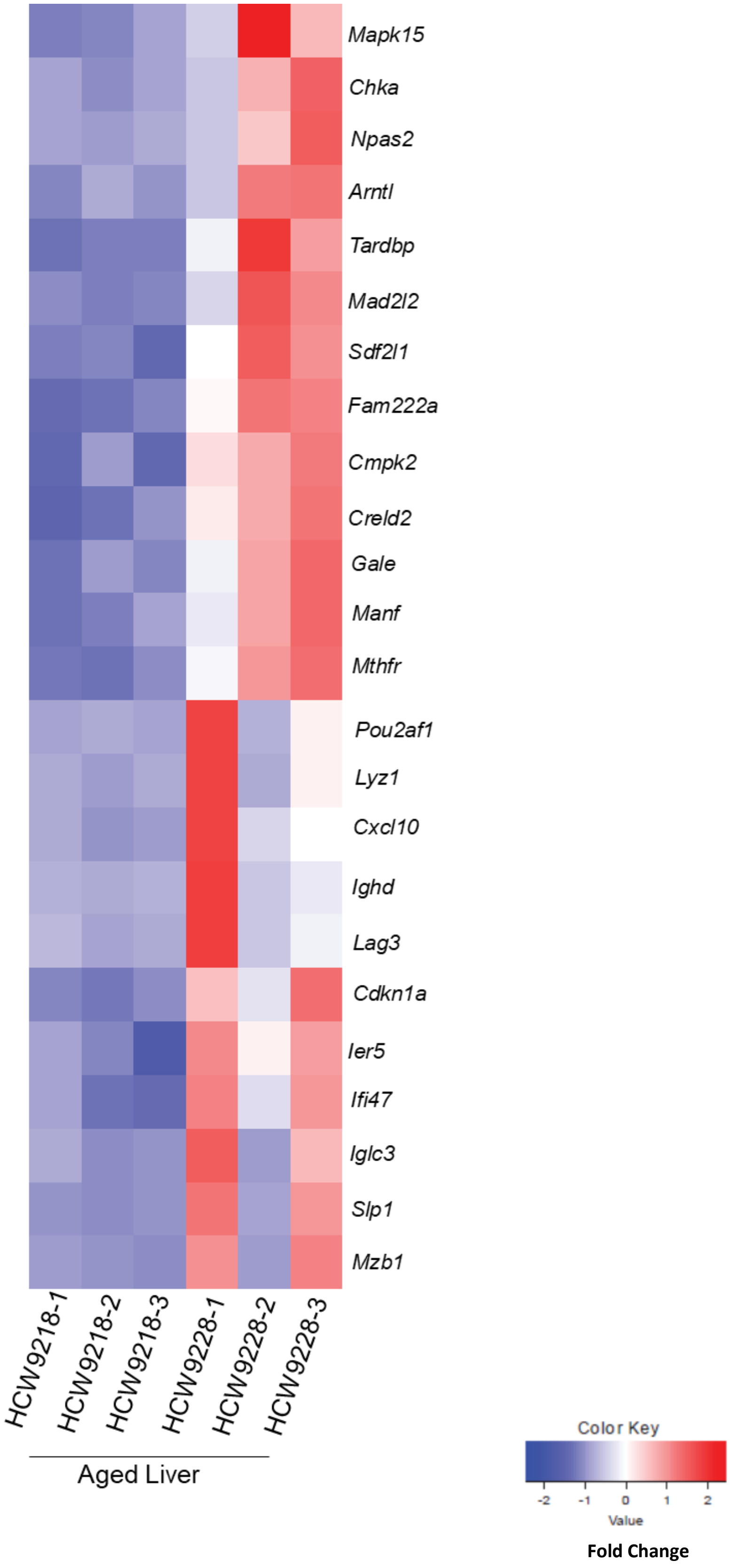

Fig. S7. Expression of representative enriched genes Albumin(*Alb)* (A) Transthyretin(*Ttr)* (B) Tyrosine Aminotransferase(*Tat)* (C) and Serine Peptidase Inhibitor, Clade A, Member 3K(*Serpina3k)* (D) for Hepatocyte cell type*s.* Gene expression violin plots were shown in log-scale UMI. Colors correspond to cluster and treatment .

A.

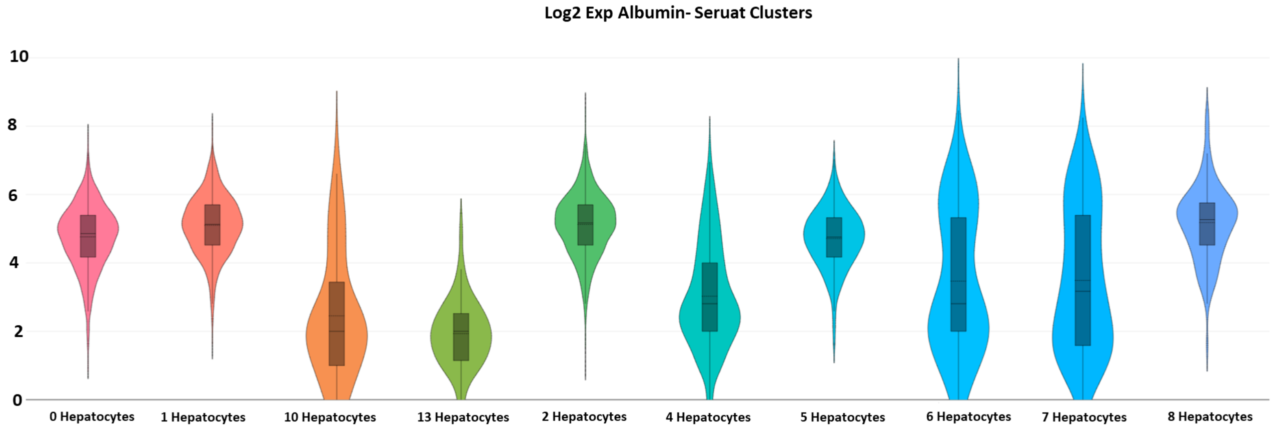

B.

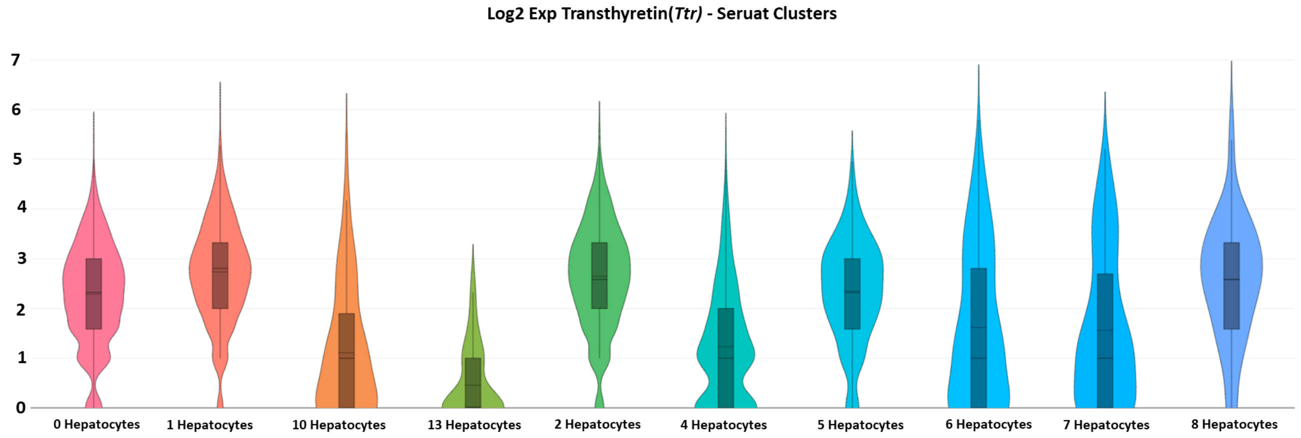

Fig. S**7**. Cont. Expression of representative enriched genes Albumin(*Alb)* (A) Transthyretin(*Ttr)* (B) Tyrosine Aminotransferase(*Tat)* (C) and Alpha 2-HS Glycoprotein (*Ahsg)*- (D) for Hepatocyte cell type. Gene expression violin plots were shown in log-scale UMI. Colors correspond to cluster and treatment .

C.

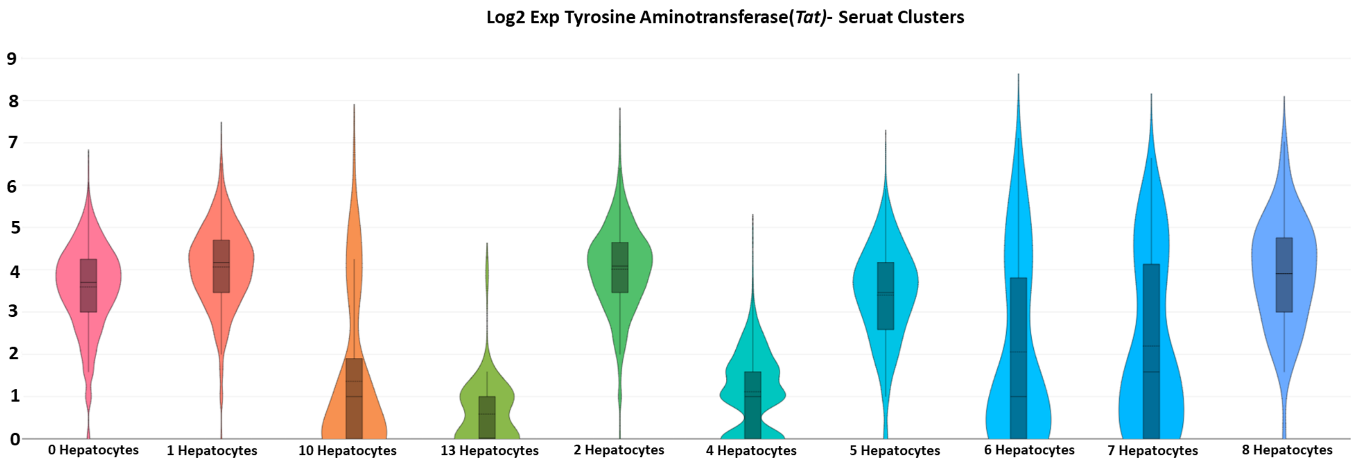

D.

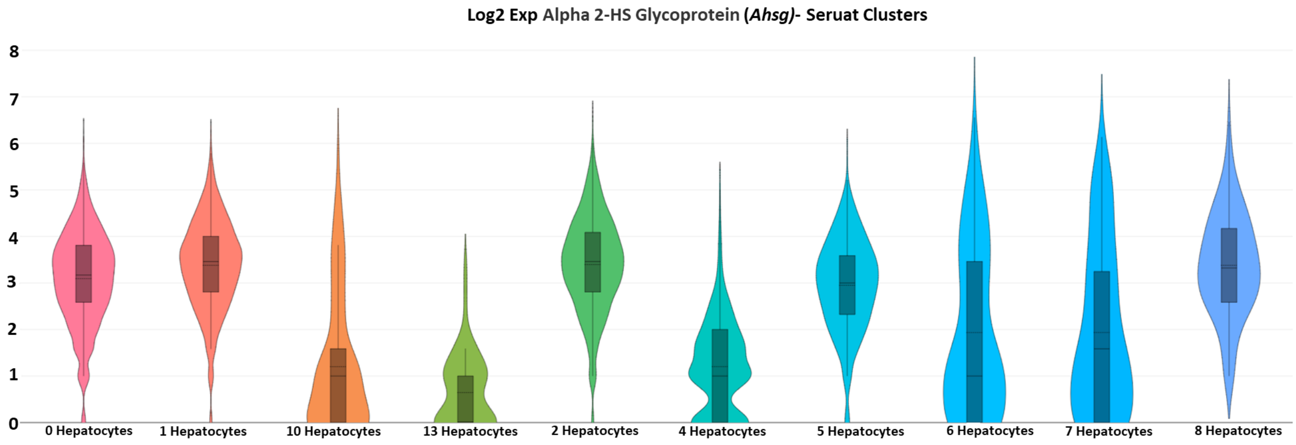

Fig. S8. Expression of representative enriched gene C-type lectin domain family 4, member g (*Clecl4g)* (A) Kinase Insert Domain Protein Receptor (*Kdr)* (B) and Fatty Acid Binding Protein 4, Adipocyte (*Fabp4)* (C) for Endothelial cell type. Gene expression violin plots were shown in log-scale UMI. Colors correspond to cluster and treatment .

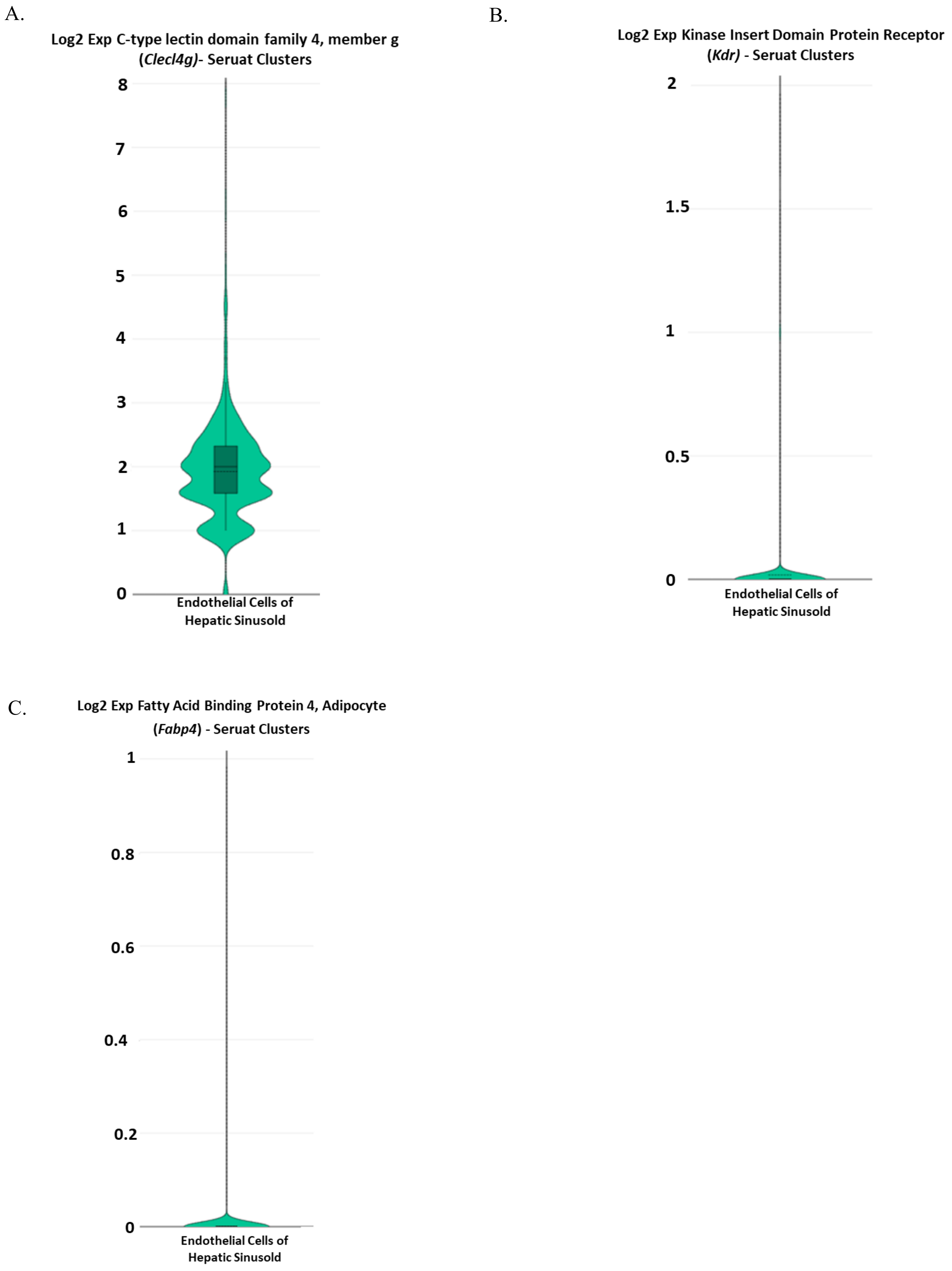

Fig. S9. Behavioral study indicate minimal acute significant performance maintenance by both HCW9218 and HCW9228 in naturally aged mice. (A-C)(A) Grip strength, (B) Rotarod test and (C) Open field test performed in aged mice treated with HCW9218 and HCW9228 to measure acute effect on the peak force value 30 days after the first dose of each respective treatment . (n=5 for Control, HCW9218, and HCW9228; *p* values were determined by ordinary two-way ANOVA with Tukey's multiple comparisons test where there are more than two groups.

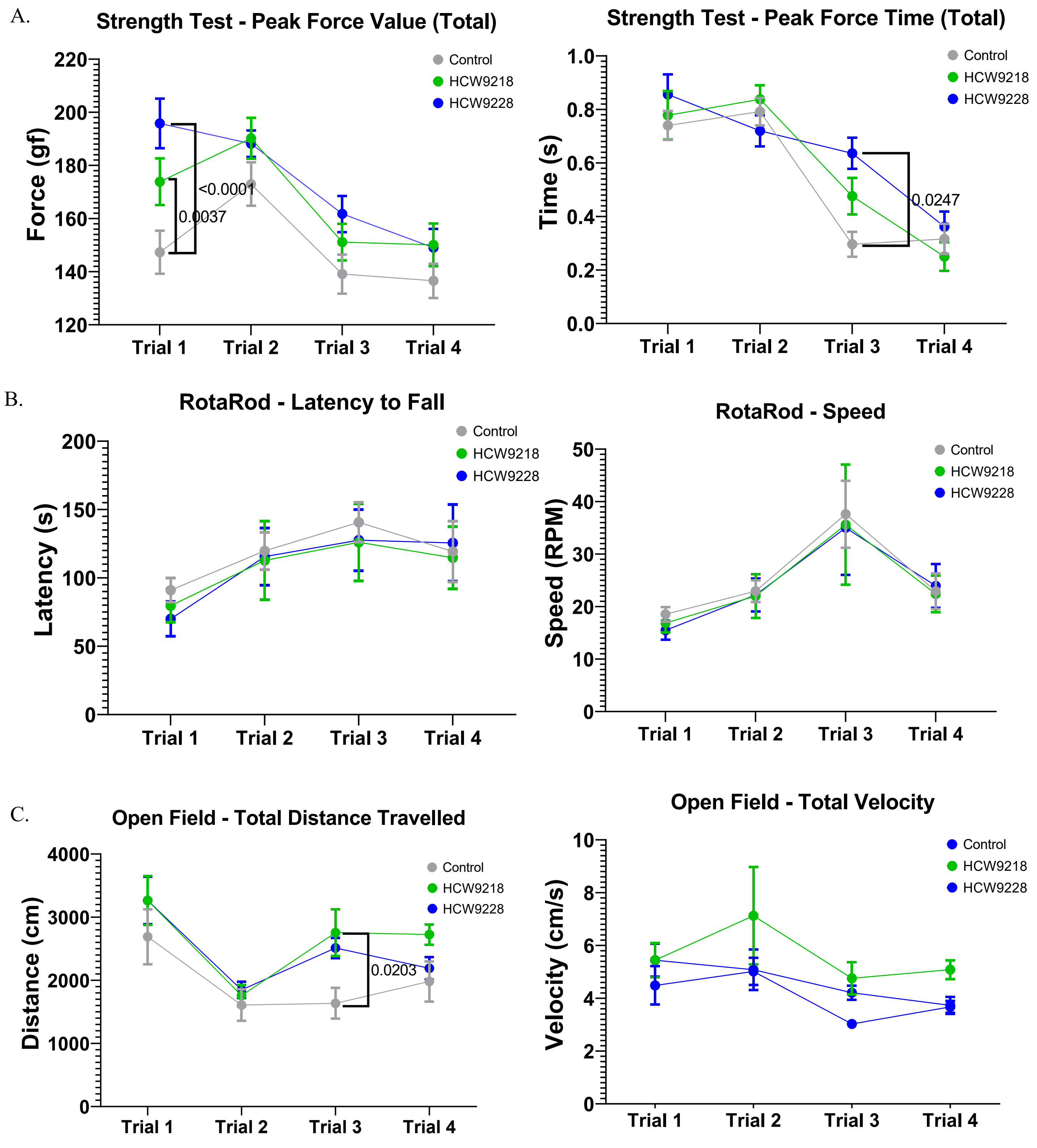

Table S3. CyTOF panels list of antibodies

CyTOF mAbs				
Channel	Antibody	Clone	Source	Identifier
089Y	CD45	30-F11	Fluidigm	3089005B, RRID:AB_2651152
141Pr	Ly6G	1A8	Fluidigm	3141008B, RRID:AB_2814678
142Nd	EOMES	W17001A	BioLegend	157702, RRID:AB_2876538
143Nd	CD11b	M1/70	Fluidigm	3143015B, RRID:AB_2811240
144Nd	CD16/32	93	Fluidigm	3144009B, RRID:AB_2814674
145Nd	CD69	H1.2F3	Fluidigm	3145005B
146Nd	p21	F-5	Santa Cruz Bio	sc-6246, RRID:AB_628073
147Sm	CD200R	OX-110	BioLegend	123902, RRID:AB_1227747
148Nd	CENP-B	NA	AbCam	ab25734, RRID:AB_726801
149Sm	CD19	6D5	Fluidigm	3149002B, RRID:AB_2814679
150Nd	CD27	LG.3A10	Fluidigm	3150017B
151Eu	CD25	3C7	Fluidigm	3151007B, RRID:AB_2827880
152Sm	CD3e	145-2C11	Fluidigm	3152004, RRID:AB_2687836
153Eu	NKG2A	16A11	BioLegend	142802, RRID:AB_11124347
154Sm	CTLA-4	UC10-4B9	Fluidigm	3154008B
155Gd	KLRG-1	2F1	BD Biosciences	562190, RRID:AB_11154418
156Gd	CD122	5H4	BioLegend	105902, RRID:AB_313225
158Gd	FoxP3	FJK-16s	Fluidigm	3158003A, RRID:AB_2814740
159Tb	PD-1	RMP1-30	Fluidigm	3159006B
160Gd	CD62L	MEL-14	Fluidigm	3160008, RRID:AB_2687840
161Dy	Tbet	4B10	Fluidigm	3161014B, RRID:AB_2858233
162Dy	Ki-67	B56	Fluidigm	3162012B, RRID:AB_2888928
163Dy	CD28	37.51	BioLegend	102119, RRID:AB_2563736
164Dy	CCR7	4B12	Fluidigm	3164013A, RRID:AB_2814683
165Ho	NK1.1	PK136	Fluidigm	3165018B
166Er	p16	NA	Proteintech	10883-1-AP, RRID:AB_2078303
167Er	NKp46	29A1.4	Fluidigm	3167008B
168Er	CD8	53-6.7	Fluidigm	3168003B, RRID:AB_2811241
169Tm	TCR b	H57-597	Fluidigm	3169002B, RRID:AB_2827883
170Er	CD49b	HMa2	Fluidigm	3170008B, RRID:AB_2814741
171Yb	CD44	IM7	Fluidigm	3171003B
172Yb	CD4	RM4-5	Fluidigm	3172003B, RRID:AB_2811242
173Yb	GranzymeB	GB11	Fluidigm	3173006B, RRID:AB_2811095
174Yb	LAG-3	C9B7W	Fluidigm	3174019B
175Lu	CD127	A7R34	Fluidigm	3175006B
176Yb	B220	RA3-6B2	Fluidigm	3176002B
209Bi	CD11c	N418	Fluidigm	3209005B, RRID:AB_2811244
