# Supplementary material for: Immunotherapeutic approach to reduce senescent cells and alleviate senescence‐associated secretory phenotype in mice
Source: Aging Cell. 2023 Mar 26;22(5):e13806. doi: 10.1111/acel.13806 (PMC10186597; doi:10.1111/acel.13806)
Supplement: Supplementary file 2 — AppendixS2 [file ACEL-22-e13806-s001.docx]

Methods Section

**Immunotherapeutic approach to reduce senescent cells and alleviate senescence-associated secretory phenotype in mice**

Niraj Shrestha^†1^, Pallavi Chaturvedi^†1^, Xiaoyun Zhu^†1^, Michael Dee^1^, Varghese George^1^, Christopher Janney^1^, Jack Egan^1^, Bai Liu^1^, Mark Foster^2^, Lynne Marsala^2^, Pamela Wong^2^, Celia C. Cubitt^2^, Jennifer A. Foltz^2^, Jennifer Tran^2^, Timothy Schappe^2^, Karin Hsiao^3^, Gilles M. Leclerc^1^, Lijing You^1^, Christian Echeverri^1^, Catherine Spanoudis^1^, Ana Carvalho^1^, Leah Kanakraj^1^, Crystal Gilkes^1^, Nicole Encalada^1^, Lin Kong^1^, Meng Wang^1^, Byron Fang^1^, Zheng Wang^1^, Jin-an Jiao^1^, Gabriela Muniz^1^, Emily K. Jeng^1^, Nicole Valdivieso^1^, Liying Li^1^, Richard Deth^3^, Melissa M. Berrien-Elliott^2^, Todd A. Fehniger^2^, Peter Rhode^1^ and Hing C. Wong^1‡^

Correspondence to: [HingWong@hcwbiologics.com](mailto:HingWong@hcwbiologics.com)

Methods

**Animals and experimental protocols**

Five-week-old male db/db mice [BKS.Cg-*Dock7^m^* +/+ *Lepr^db^*/J (Wildtype for *Dock7^m^*, Homozygous for *Lepr^db^*), strain#000642] from Jackson Laboratory (Bar Harbor, ME) were fed with standard chow diet (Irradiated 2018 Teklad global 18% protein rodent diet, Envigo, IN) and received drinking water ad libitum. Six and 76-week-old female C57BL/6J mice were purchased from Jackson Laboratory (Bar Harbor, ME). The mice (n = 5-8) were subcutaneously injected with one or two doses of either PBS, HCW9218 (3 mg/kg), or HCW9228 (3 mg/kg) as indicated in the studies. Depending upon the experiments, mice were euthanized at either day 4, day 10, day 60, or day 120. For short-term experiments (day 4 and day 10), the spleen and kidney were harvested. For long-term experiments (day 60 and day 120), spleen were harvested. All animal studies were performed according to HCW Biologics animal care guidelines under Institutional Animal Care and Use Committee (IACUC)-approved protocols. For survival studies, 76-week-old male and female C57BL/6J mice (10 mice each group) were subcutaneously injected with either PBS or HCW9218 at 3 mg/kg and survival was analyzed by Kaplan–Meier estimates and the log-rank statistic. A toxicology studies was conducted on 7-week-old female and male C57BL/6 mice (Medicilon Preclinical Research, Shanghai, China) as described below.

**Lymphocyte staining of peripheral blood and spleen**

Peripheral blood (submandibular vein) and single cell suspension of splenocytes were stained with antibodies (Biolegend, San Diego, CA) for immune cell-surface expression of CD45, CD3, CD8α, CD4, NK1.1, CD25, Nkp46, CD44, CD62L, CD127 for 10 minutes (min) at room temperature (RT) following red blood cells lysis. After surface staining, cells were washed in FACS buffer (1X PBS (Hyclone, Utha) containing 0.5% BSA (EMD Millipore, MA)) and 0.001% sodium azide (Sigma, MO), centrifuged at 1,500 RPM for 5 min at RT, and incubated in fixation buffer (Invitrogen, MA) for 20 min at 4 °C. Then, cells were washed with permeabilization buffer (Invitrogen, MA), and stained for the intracellular markers Foxp3, Ki67, and Granzyme B (Biolegend) for 30 min at RT. Stained samples were analyzed by flow cytometry (Celesta-BD Bioscience, CA) using the FlowJo software version 5.1. The antibodies are listed in the Supplement Table S3.

**Cytotoxicity assay**

Spleens from mice subcutaneously injected with either PBS, HCW9218 (3 mg/kg) or HCW9228 (3 mg/kg) (five mice per group) were collected either at day 2 or day 4 and single-cell suspension prepared as described previously^1^. Splenocytes were stained with Cell Trace Violet (Thermofisher Scientific, MA) and mixed with Yac1 cells at different Effector: Target (E:T) ratios and killing was evaluated after 16 hrs. Following incubation, cells were washed in 1X PBS and resuspended in complete media with propidium iodide (PI) and killing was assessed by flow cytometry (FACS Celesta, BD Bioscience) and analyzed with FlowJo software version 5.1.

**αCD3/αCD28 stimulation assays**

For stimulation assays, splenocytes were harvested from young and aged mice subcutaneously injected with either PBS, HCW9218 (3 mg/kg), or HCW9228 (3 mg/kg) (5 mice per group) either at day 2 or day 4. Single cells suspension of splenocytes were prepared as described previously ^1^, and plated at 2 x 10^5^ cells per well in the 96-well U-bottom plate (Costar). Cells were stimulated with the Milteyni T Cell Activation/Expansion Kit (Cat# 130-093-627, Milteyni) at a 1:1 ratio of beads to cells and cultured for 4 days. Then, supernatants were collected to measure TNF-α or IFN-γ cytokine released using the Luminex MAGPIX multiplexing cytokine assay (Thermo Fisher Scientific, MA).

**Determination of plasma insulin and glucose**

After 12 hrs of fasting, blood was collected by submandibular vein puncture. Plasma insulin and glucose were determined using either the Crystal Chem ultra-sensitive mouse insulin ELISA kit (Cat# 90080, Crystal Chem, Elk Grove Village, IL), a OneTouch UltraMini Blood Glucose Meter (SN# LKH03D1ER, LifeScan, Malvern, PA) or the Crystal Chem mouse glucose assay kit (Cat# 8692, Crystal Chem, Elk Grove Village, IL), following manufacturer’s instructions. The insulin resistance index (HOMA-IR) was calculated according to the formula: fasting insulin (microU/L) x fasting glucose (nmol/L)/22.5.

**Assessment of immune cell metabolic activities**

Extracellular acidification rates (ECAR) and oxygen consumption rates (OCR) of splenocytes from db/db, aged and young mice were measured as described previously ^2^ at day 2, 4, or 90 depending upon studies.

**Plasma cytokine assays**

Blood was collected from the submandibular vein in tubes containing EDTA and plasma was isolated by centrifugation. The plasma TGF-β levels were analyzed by using cytokine array, TGF-β 3-plex (TGF-β 1-3) (Eve Technologies, Calgary, AL, Canada). For pro-inflammatory cytokine expression, plasma samples were diluted in PBS and analyzed using a Mouse Cytokine Array Proinflammatory Focused 10-plex (MDF10) (Eve Technologies, Calgary, AL, Canada).

**RNA isolation, cDNA synthesis, and quantitative PCR**

For quantitative PCR (qPCR), total RNA was isolated from snap frozen tissues and homogenized in Trizol using bead mill homogenizer (Fisher Scientific, MA). After homogenization, total RNA was extracted using RNeasy kit (Qiagen, CA). One microgram of total RNA was used for first-strand DNA synthesis using the QuantiTech reverse transcription kit (Qiagen, CA). RT-qPCR was performed using the mouse specific primers and Taqman gene expression master-mix (Thermo Fisher Scientific, MA). qPCR was performed using QuantStudio 3 Real-Time PCR System (Applied Biosystems, MA). The expression of each target mRNA relative to 18S rRNA was calculated based on the threshold cycle (Ct) as 2^−Δ(ΔCt)^, where ΔCt = Ct_target_ − Ct_18S_ and Δ(ΔCt) = ΔCt_test_ − ΔCt_control_. The FAM-labeled mouse specific primers were purchased from Thermo Fisher Scientific. Primers for real-time PCR are listed in Primer List Table. The indices for presentation of the qPCR data were generated as follows: Aging index: Mean of normalized values of *Cdkn1a, Cdkn2a, Bambi* and *Igf1r*; SASP Index: Mean of normalized values of *Il1a, Il1b, Il6*, *Ccl2*, and *Tnfa*; β cell Index: Mean of normalized values of *Ins1, Gck, Mafa, Pdx1, Neurod1, and Nkx6-1*. The normalized values were calculated as follows: the normalized PBS group value = PBS group value / PBS group value, and the normalized HCW9218 group value = HCW9218 group value/ PBS group value.

**Determination of glucose**

Approximately 100 μL of blood was collected via a submandibular vein from conscious mouse. Ten microliters of blood were used to measure Glucose (Crystal Chem) according to the manufacturer’s instructions.

**Immunohistochemistry**

For immunohistochemistry of pancreas, the db/db mice were euthanized 4 weeks after the 2^nd^ dose injection, and pancreas were removed *en bloc*, immersion-fixed in 4% formaldehyde (4% formaldehyde in 0.1M phosphate buffer; PBS pH 7.4) and stored at 4 ^o^C until processing. Then, dissected pancreas were paraffinized, embedded, and sectioned at the Department of Pathology, University of Miami Miller School of Medicine.

Through our collaboration with the Human Immune Monitoring Shared Resource (HIMSR) at the University of Colorado School of Medicine, we performed multispectral imaging using the Akoya Vectra Polaris instrument. This instrumentation allows for phenotyping, quantification, and spatial relationship analysis of tissue infiltrate in formalin-fixed paraffin-imbedded biopsy sections. Briefly, the slides were deparaffinized, heat treated in antigen retrieval buffer, blocked, and incubated with rabbit primary antibodies against insulin (1:100, Cat# 4590, Cell Signaling Technology, MA) and p21 (1:50, EPR362, Abcam, MA), followed by horseradish peroxidase (HRP)-conjugated secondary antibody polymer (anti-rabbit), and HRP-reactive OPAL fluorescent reagents [OPAL-520 for insulin (1:150) and OPAL-570 for p21(1:50), Akoya, MA]that use TSA chemistry to deposit dyes on the tissue immediately surrounding each HRP molecule. To prevent further deposition of fluorescent dyes in subsequent staining steps, the slides were stripped in between each stain with heat treatment in antigen retrieval buffer (Citrate buffer for insulin and EDTA buffer for p21). The whole slide scans were collected with the Akoya Vectra Polaris instrument using the 20x objective with a 0.5-micron resolution. For pancreas, 3-color images were analyzed with inForm software (Akoya, MA) to unmix adjacent fluorochromes, subtract autofluorescence, segment insulin+ regions of the tissue, compare the frequency and location of cells, segment cellular cytoplasmic and nuclear regions, and phenotype infiltrating cells according to cell marker expression. For all tissues analyzed, quantification of immunostaining was performed from 6 high power fields per section/mouse, with investigators blinded to treatment assignment. Representative images were selected to represent the mean value of each condition.

**Bulk RNA sequencing (RNA-seq)**

Tissue samples were homogenized using bead mill homogenizer in 1 mL of Trizol (Thermo Fisher, MA) and transferred in centrifuge tubes. Total RNA was extracted using the RNeasy Mini Kit (Qiagen, CA) according to the manufacturer's instructions.

Library preparations, sequencing reactions and bioinformatic analysis were conducted at GENEWIZ, LLC. (South Plainfield, NJ). Briefly, RNA sequencing libraries were prepared using the NEBNext Ultra II RNA Library Prep Kit for Illumina following manufacturer’s instructions (NEB, Ipswich, MA). For this, mRNAs were first enriched with Oligo (dT) beads, and fragmented for 15 min at 94 °C. First strand and second strand cDNAs were subsequently synthesized. cDNA fragments were end repaired and adenylated at 3’ends, and universal adapters were ligated to cDNA fragments, followed by index addition and library enrichment by limited-cycle PCR. The sequencing libraries were validated on the Agilent TapeStation (Agilent Technologies, Palo Alto, CA), and quantified by using Qubit 2.0 Fluorometer (Life Technology, Carlsbad, CA) as well as by quantitative PCR (KAPA Biosystems, Wilmington, MA). The sequencing libraries were clustered on 1 flowcell lane. After clustering, the flowcell was loaded on the Illumina HiSeq instrument (4000 or equivalent) according to manufacturer’s instructions. The samples were sequenced using a 2 x 150bp Paired End (PE) configuration. Image analysis and base calling were conducted by the HiSeq Control Software (HCS). Raw sequence data (.bcl files) generated from Illumina HiSeq were converted into fastq files and de-multiplexed using Illumina's bcl2fastq 2.17 software. One mismatch was allowed for index sequence identification. Sequence reads were trimmed to remove possible adapter sequences and nucleotides with poor quality using Trimmomatic ver.0.36. The trimmed reads were mapped to the Mus musculus GRCm38 reference genome available on ENSEMBL using the STAR aligner ver.2.5.2b. The STAR aligner is a splice aligner that detects splice junctions and incorporates them to help align the entire read sequences. BAM files were generated as a result of this step. Unique gene hit counts were calculated by using feature counts from the Subread package ver.1.5.2. The hit counts were summarized and reported using the gene id feature in the annotation file. Only unique reads that fell within exon regions were counted. If a strand-specific library preparation was performed, the reads were strand-specifically counted. After extraction of gene hit counts, the gene hit counts table was used for downstream differential expression analysis. Using DESeq2, a comparison of gene expression between the customer-defined groups of samples was performed. The Wald test was used to generate p-values and log2 fold changes. Genes with an adjusted p-value < 0.05 and absolute log2 fold change > 1 were called as differentially expressed genes for each comparison. A gene ontology (“GO”) analysis was performed on the statistically significant set of genes by implementing the software GeneSCF ver.1.1-p2. The mgi GO list was used to cluster the set of genes based on their biological processes and determine their statistical significance. To estimate the expression levels of alternatively spliced transcripts, the splice variant hit counts were extracted from the RNA-seq reads mapped to the genome. Differentially spliced genes were identified for groups with more than one sample by testing for significant differences in read counts on exons (and junctions) of the genes using DEXSeq. For groups with only one sample, the exon hit count tables were provided.

The DEGs (“Differentially Expressed Genes”) obtained from RNA-Seq-based expression profiling were analyzed by using iDEP (integrated Differential Expression and Pathway analysis) online tools ^3^ and KEGG (Kyoto Encyclopedia of Genes and Genomes, Japan) pathway was applied to analyze the function of DEGs.  KEGG is a knowledge base for systematic analysis of gene functions comprising a series of genome and enzymatic approaches and genomic information with higher order functional information ^4^, which is used for systematic analysis of gene functions and related high-level genome functional information of DGEs. The RNA-seq data presented in the study were deposited to SRA with Accession number PRJNA927323. Rest of bulk RNA-seq and the single cell RNA-seq data are submitted as supplementary data file

**Nuclei isolation and snRNA-Seq library preparation and sequencing from frozen tissue**

To further examine the long-term impacts of HCW9218 treatments on hepatocytes from naturally-aged mice, liver tissues obtained from mice collected 60 days after the second treatment were subjected to single-cell RNA library construction and sequencing. We isolated nuclei from frozen mice liver tissue samples and constructed 3’ single cell gene expression libraries (Next GEM ver. 3.1) using the 10x Genomics Chromium system. The libraries were sequenced with ~200 million PE150 reads per sample on Illumina NovaSeq. The sequencing reads were analyzed with mouse reference genome mm10 using Cell Ranger ver.7.0.0. Introns were included in the analysis. Aggregation of the samples was also performed, normalizing for the total number of confidently mapped reads across libraries. Using Loupe Browser, we filtered out nuclei with >5% mitochondria (2% of the barcodes were filtered out). This resulted in 4942 nuclei from HCW9218-treated, and 4669 nuclei from PBS-treated (control) samples. We re-clustered the barcodes in 5 different clusters after filtering with Loupe Browser and did cell typing with SciBet (Li, C *et al.*  *Nat Commun* **11**, 1818 (2020). All of the clusters were identified as hepatocytes subtypes (Fig. S5A-D), endothelial cells (Fig. S7A-C) and leukocytes (Fig. S7A-D) based on previously published markers. We marked the results in the Loupe Browser under the “mito<5%” category, and “mito<5%” UMAP. The differential expressions of all significant genes and just the highly expressed genes were calculated with the Loupe Browser.

**Mass cytometry**

All mass cytometry data were collected on a CyTOF2 mass cytometer (Fluidigm) and analyzed using Cytobank. Mass cytometry data were analyzed using previously described methods. For detailed information, refer to the Supplementary Materials and Methods (*Becker-Hapak et al*, Cancer Immunology Research, 2021 Sep;9(9):1071-1087). Diversity was assessed on eight individual mice at baseline (cells from liver and spleen), after mice subcutaneously injected with either PBS, HCW9218 (3 mg/kg), or HCW9228 (3 mg/kg) (5 mice per group) were collected either at day 4 or day 10.

**Tissue ELISA**

IL-1α, IL-1β, Collagen, IL-6, TNF-α and IL-8 (R&D Systems), Fibronectin, Collagen (Abcam, MA), were evaluated in liver tissue homogenates by using a mouse ELISA kit according to the manufacturer’s instructions (R&D Systems, MN).

**Motor function test**

For behavioral experiments, the aged C57Bl/6J mice were housed in the behavior room on a reversed 12 hrs light/dark cycle (7 AM – 7 PM). Mice were moved to the behavior room 10 days prior to beginning of behavioral experiments to acclimate to the change in light cycle and were handled for at least 5-10 min daily to help reduce the stress of handling during behavioral experiments. The behavior room was maintained at a temperature of between 20 °C and 23.8 °C, and consistent white noise was used in the behavior room to reduce stress and maintain a constant ambient environment. Mice were given ad libitum access to food and water, and all behavioral experiments were performed between the hours of 9 AM and 5 PM. Unless otherwise stated, all tests were run in red light at approximately 3-4 lux. A battery of three motor behavior tests were run to measure strength, coordination, and ambulation. Tests were run at four (4) timepoints: 30 days post dose 1 (Trial 1), 3-6 days post dose 2 (Trial 2), 30 days post dose 2 (Trial 3), 60 days post dose 2 (Trail 4).

**Grip strength**

Using the Ugo Basile Grip Strength Meter (Stoelting) with the grid for combined front and hind limb grip strength, objective neuromuscular performance was measured in terms of peak force generated (gf) and amount of time to hold the peak force (peak force time(s)). Mice were measured for five repetitions each timepoint to determine an average for each mouse, which was used as the score for that trial. Calculations were completed via GraphPad Prism 9.3.1.

**Rotarod**

The Ugo Basile Mouse Rota-Rod (Stoelting) apparatus was used to measure motor performance, coordination, and learning. Prior to running tests, mice were placed on the device and allowed to acclimate and explore by walking at 5 rotation per minute (RPM) for 5 min. On testing days, mice were placed on the rotarod at 5 RPM. Once all mice were placed in their lanes (total of 5 mice per group), the test was started. Speed was increased from 5 RPM to 50 RPM over 300 seconds (5 min). Time and speed for each mouse was collected when either a mouse dropped from the rod or made one full rotation gripping the rod. Once the test ended for all mice running together, mice were removed, placed back in home cage, and the apparatus was cleaned with 70% EtOH. The test was then repeated for the next group, and this process was repeated for a total of three times per group per timepoint, providing a 10–15 minute break for each group between each run. All times and speeds for all repetitions for each group were combined to determine average latency to fall and speed (RPM) for each timepoint. Calculations were completed via GraphPad Prism 9.3.1.

**Open field**

Open field experiments were setup, video was recorded, and behavior scored via Novus EthoVision XT (ver.15.0.1416). The open field apparatus consisted of a black acrylic box (40 cm x 40 cm x 30 cm) with a gray base (MazeEngineers, Conduct Science). Tests were run in a combination of indirect red and white (3200K, 10%) light at a total of approximately 16 lux inside the open field apparatus. Mice were placed in the center of the apparatus and allowed to explore freely for ten 10 minutes. Distance travelled and speed were measured. All mice were allowed a single exploration of the open field at each time point. Calculations were completed via GraphPad Prism 9.3.1.

**Toxicology**

An IACUC-approved study protocol was performed under Good Laboratory Practice guidelines (with appropriate Quality Assurance review) to evaluate the toxicological effects of multidose administration of HCW9218 in C57BL/6 mice (Medicilon Preclinical Research, Shanghai, China). Animals (7 weeks old, 30 animals/sex/group) were treated every 14 days for two doses (study day (SD) 1 and 15) with 5, 25, or 100 mg/kg HCW9218 or PBS administered subcutaneously. Throughout the in-life study phase, animals were assessed for cage-side, clinical and behavioral changes, food consumption, body weight, injection site changes, and ophthalmic function. Blood was collected for hematology, chemistry, and coagulation analyses on SD22 or SD29, and urine samples were collected for urinalyses on the day before blood collection. Clinical pathology assessments, including physical examination, gross necropsy, organ weight measurements, and histopathology, were performed 7 days (SD22) and 2 weeks (SD29) after treatment. Based on these endpoints, the No Observed Adverse Effect Level (NOAEL) of HCW9218 in this study was considered to be 100 mg/kg.

### Statistical analysis

GraphPad Prism (Ver. 9.3.1) was used to generate graphs and for statistical analysis. Details on sample size (biological replicates), number of repetitions, and statistical tests are listed in Figure Legends. Accordingly, Student’s *t* test or Mann-Whitney *U* test was used to determine statistical significance between two groups. One-or two-way analysis of variance (ANOVA) with Tukey’s multiple comparison test was used to determine significant differences between multiple groups.  Only significant statistic differences (p < 0.05) were displayed in the Figures. Flow cytometry data were analyzed using FlowJo software (v10.6; Tree Star).

**References**

1 Liu, B. *et al.* Bifunctional TGF-beta trap/IL-15 protein complex elicits potent NK cell and CD8(+) T cell immunity against solid tumors. *Mol Ther* **29**, 2949-2962, doi:10.1016/j.ymthe.2021.06.001 (2021).

2 Chaturvedi, P. *et al.* Immunotherapeutic HCW9218 augments anti-tumor activity of chemotherapy via NK cell-mediated reduction of therapy-induced senescent cells. *Mol Ther* **30**, 1171-1187, doi:10.1016/j.ymthe.2022.01.025 (2022).

3 Ge, S. X., Son, E. W. & Yao, R. iDEP: an integrated web application for differential expression and pathway analysis of RNA-Seq data. *BMC Bioinformatics* **19**, 534, doi:10.1186/s12859-018-2486-6 (2018).

4 Kanehisa, M. & Goto, S. KEGG: kyoto encyclopedia of genes and genomes. *Nucleic Acids Res* **28**, 27-30, doi:10.1093/nar/28.1.27 (2000).
